# Schrödinger Operators with Multiple Aharonov–Bohm Fluxes

**DOI:** 10.1007/s00023-024-01446-x

**Published:** 2024-05-25

**Authors:** Michele Correggi, Davide Fermi

**Affiliations:** 1 Dipartimento di Matematica, Politecnico di Milano, P.zza Leonardo da Vinci, 32, 20133 Milan, Italy; 2 Istituto Nazionale di Fisica Nucleare, Sezione di Milano, Milan, Italy

**Keywords:** 47A07, 47A40, 81Q10, 81Q70, 81U99

## Abstract

We study the Schrödinger operator describing a two-dimensional quantum particle moving in the presence of $N \geqslant 1$ Aharonov–Bohm magnetic fluxes. We classify all the self-adjont realizations of such an operator, providing an explicit characterization of their domains and actions. Moreover, we examine their spectral and scattering properties, proving in particular the existence and completeness of wave operators in relation with the free dynamics.

## Introduction and Main Results

Back in 1959, Aharonov and Bohm [5] predicted that the presence of a magnetic field would induce a phase shift in the wave function of a charged quantum particle, even if the particle is confined for all times to a space region where the magnetic field vanishes identically. Analogous theoretical considerations had previously been advanced by Ehrenberg and Siday [32]. Undisputed experimental evidence of the Aharonov–Bohm (AB) effect was provided in 1986 by Tonomura et al. [79], who used micro-sized toroidal magnets coated in superconducting layers to minimize leakages of the magnetic field and thus realize perfect shielding for the electron wave function. Though a controversy about the fundamental interpretation of the AB effect somehow persists even nowadays [43, 54], the reality of the physical phenomenon is by now unquestionable. In fact, it appears to play a prominent role in many areas of condensed matter physics. For example, sharply localized fluxes of AB-type occur in *type-II superconductors*, when strong magnetic fields pierce through an almost 2D layer [3]. Another prominent instance regards *anyons*, quasi-particles excitations of 2D electron gases in the fractional quantum Hall regime which carry fractional charge and obey fractional statistics [10, 48, 59, 80]. Notably, with a suitable gauge fixing, anyons can be described in terms of bosonic wave functions with 2-body AB interactions. Yet another research line, which is recently attracting attention, is that of *AB cages*, where the destructive interference produced by singular magnetic fluxes provides a localization mechanism for non-interacting particles [14, 58].

With the above applications in mind, we consider a prototype model including a charged scalar particle and *N* parallel ideal solenoids, each of infinite length and zero diameter like in the original AB setting. After factorization of the axial direction, the dynamics of the particle is determined by a Schrödinger operator in $\mathbb {R}^2$ of the form

1.1 $$\begin{aligned} \left( - \,i \nabla + \sum _{n \,=\, 1}^{N}\alpha _n\; \frac{\left( \textbf{x}- \textbf{x}_n \right)^{\perp }}{|\textbf{x} - \textbf{x}_{n}|^2} \right) ^{\!2}. \end{aligned}$$

Here, $\textbf{x}= (x,y) \in \mathbb {R}^2$ and $\textbf{x}^{\perp } = (- y, x)$, while $\textbf{x}_n$ and $\alpha _n \in \mathbb {R}$ identify the positions of the solenoids and the associated magnetic fluxes, respectively. It is worth noting that the magnetic field matching the vector potential in (1.1) is formally given by a sum of Dirac delta distributions, namely,

$$\begin{aligned} B(\textbf{x}) = \sum _{n \,=\, 1}^{N}\, 2\pi \,\alpha _n\,\delta (\textbf{x} - \textbf{x}_{n}). \end{aligned}$$

Hence, *B* has compact support and we can reasonably expect that scattering w.r.t. the free Laplacian is well defined. In light of the above physical interpretation, we may also think of the formal operator introduced above as describing in suitable regimes a tracer particle moving in a gas of *N* anyons with heavy masses (or two-dimensional quasi-particles carrying localized magnetic fluxes), so that the latters may be modeled by fixed AB fluxes at the particles’ positions $\textbf{x}_n$, $n = 1, \dots , N$ [17, 20, 21, 39, 50–52, 55].

The configuration with just one solenoid has an evident rotation symmetry and, as a consequence, it can be explicitly analyzed by decomposition in angular harmonics. Building on this, an exhaustive classification of all self-adjoint realizations in $L^2(\mathbb {R}^2)$ of the Schrödinger operator (1.1) with $N = 1$ was first derived using Kreĭn-von Neumann theory by Adami and Teta [4] (see also [23] for a similar result, [15, 25–29] for more details on the radial operators and [21, 56, 62] for connections with 2-anyons systems). These single-flux Hamiltonians include the Friedrichs realization and a 4-parameter family of singular perturbations thereof, describing *s-wave* and *p-wave* zero-range interactions. The spectral and scattering properties of these operators were further investigated in [63, 71, 74, 83]. General results on the scattering matrix and total cross section for Schrödinger operators related to the single-flux Friedrichs Hamiltonian were derived in [70, 81, 82] by means of stationary representation formulas and pseudo-differential methods.

On the other hand, the approximation in resolvent sense of the above mentioned Hamiltonians with zero-range interactions by means of non-singular electromagnetic potentials was discussed in [24, 53, 75]. These works shed some light on the idealizations understood in the original AB setup, indicating in particular that Schrödinger operators comprising zero-range potentials naturally emerge when considering resonant shielding potentials. On the other hand, the effects produced by regular magnetic perturbations have also been examined. The case of a uniform magnetic field, on top of the AB singularity, was studied in [34], again by Kreǐn-von Neumann methods. A different approach based on quadratic form techniques was first proposed in [21], and later extended in [18, 36] to encompass generic, regular magnetic perturbations. Let us also mention that, in the single-flux setting, there are classical results on Hardy-type inequalities [12, 47] and dispersive estimates [41]. Another research line regards studying the AB Hamiltonian in compact domains, examining the behavior of simple eigenvalues under variations of the flux position [1, 2] (see also [35] for similar results in configurations with many coalescing poles).

Investigating cases with more than one flux ($N \geqslant 2$) is in general a harder task, due to the lack of exact solutions. Remarkably, a series expansion for the Green function associated to a 1D array of fluxes was derived in [72, 73], by universal covering space techniques. Similar methods were employed in [37, 60] to produce some explicit formulas for the solutions of the eigenvalue problem. These results apparently support a previous conjecture of Aharonov, suggesting that an array of flux tubes would act as a repulsive barrier for low-energy particles. Again by complex analysis techniques and direct computations, an analytic expression for the scattering amplitude for two opposite AB fluxes was obtained in [11], focusing separately on the limit situations of small fluxes and small distance between the fluxes.

The (semiclassical) scattering amplitude and the presence of resonances in the regime of large separation between two arbitrary singular fluxes were instead examined in [6, 7, 42, 76, 77] by means of stationary methods. Regarding Schrödinger operators of the form (1.1) with more than two fluxes, let us mention that the leading order singularity of the wave trace and resonances for the wave propagator were studied in [84]. We also highlight that a rigorous diamagnetic inequality for Schrödinger operators of the form (1.1) was derived in [57], and used contextually to deduce Lieb–Thirring and CLR-type eigenvalue estimates. Another valuable theoretical approach to the analysis of configurations with several magnetic fluxes is the mean field approximation. Making reference to this regime, by perturbative and numerical computations, the scattering from a periodic array of fluxes was examined in [46] and the density of states in the thermodynamic limit was studied in [30, 31]. Finally, let us remark that the AB effect was also investigated for Schrödinger and Klein-Gordon dynamics with electromagnetic potentials in the exterior of compact obstacles. In particular, high-velocity estimates for the scattering operator and inverse scattering results were deduced by means of time-dependent techniques in [8, 9].

All the works on multiple fluxes mentioned above refer to the Friedrichs realization of the operator (1.1). This means that the attention is always restricted to wave functions vanishing at the points $\textbf{x}_{n}$, which amounts to consider only perfectly shielded solenoids. Nonetheless, the single-flux studies cited before give a strong indication of the fact that other self-adjoint realizations of (1.1) in $L^2(\mathbb {R}^2)$ should exist and that they cannot be neglected in some specific physical contexts. It goes without saying that a more faithful physical representation can be obtained taking also into account relativistic effects. In this regard, to describe the motion of a spin 1/2 charged particle confined to a 2D slab, punctured by ideal solenoids, some authors have considered Pauli and Dirac operators analogous to the Schrödinger one introduced in (1.1). The self-adjointness and spectral features of these operators were investigated in [13, 33, 38, 45, 64, 65], focusing especially on the degeneracy of the zero-energy modes and on the related Aharonov–Casher formula.

In this work, we first characterize all the admissible self-adjoint realizations in $L^2(\mathbb {R}^2)$ of the Schrödinger operator (1.1) using quadratic form techniques first and then applying the Kreǐn theory of self-adjoint extensions. Next, we use classical resolvent arguments to investigate the spectral and scattering properties of the Hamiltonians thus obtained.

### The Model: Schrödinger Operators with Multiple Aharonov–Bohm Fluxes

As anticipated our main goal is to study the well posedness as a self-adjoint operator of the Hamiltonian describing a quantum two-dimensional spinless particle moving in the presence of $N \geqslant 1$ AB fluxes, i.e., the formal Schrödinger operator

1.2 $$\begin{aligned} H_{N} = \left( - \,i \nabla + \textstyle \sum \limits _{n \,=\, 1}^{N}\textbf{A}_{n} \right) ^{\!2}, \qquad \textbf{A}_{n}(\textbf{x}) = \alpha _n\;\frac{(\textbf{x} - \textbf{x}_{n})^{\perp }}{|\textbf{x} - \textbf{x}_{n}|^2}, \end{aligned}$$

where, for each $n \in \{1,\dots ,N\}$, $\textbf{x}_{n} \!\in \! \mathbb {R}^2$ identifies the position of the *n*^th^ singular flux and $\alpha _n \in (0,1)$ measures its intensity (see also the next Remark 1.1). A natural dense domain where the operator above makes sense is the set $C^{\infty }_{\text {c}}(\mathbb {R}^2 {\setminus } \left\{ {\textbf {x}}_1, \dots , {\textbf {x}}_N \right\} )$, i.e., smooth functions with support away from the fluxes. Notice that the vector fields $\textbf{A}_{n}$ separately fulfill the Coulomb gauge, in the sense of Schwartz distributions:

$$\begin{aligned} \nabla \cdot \textbf{A}_{n} = 0, \qquad \forall \; n \in \{1,\dots ,N\}. \end{aligned}$$

This implies that (1.2) can be equivalently expressed on $C^{\infty }_{\text {c}}(\mathbb {R}^2 {\setminus } \left\{ {\textbf {x}}_1, \dots , {\textbf {x}}_N \right\} )$ as

$$\begin{aligned} H_{N} = - \,\Delta + 2 \left( {\sum _{n \,=\, 1}^{N}}\, \textbf{A}_{n}\right) \cdot (-i\nabla ) + \left( {\sum _{n \,=\, 1}^{N}}\, \textbf{A}_{n} \right) ^{\!2}, \end{aligned}$$

where the order of the factors in the second term on the r.h.s. is immaterial.

#### Remark 1.1

(Fluxes’ intensities). The condition $\alpha _n \in (0,1)$ for any $n \in \{1,\dots ,N\}$, rather than $\alpha _n \in \mathbb {R}$, actually entails no loss of generality, since it can always be realized via a unitary transformation. To prove this claim, assume $\alpha _n = a_n + \tilde{\alpha }_{n}$ with $a_n \in \mathbb {Z}$ and $\tilde{\alpha }_{n} \in (0,1)$, and consider the unitary maps

$$\begin{aligned} U_n: L^2(\mathbb {R}^2) \rightarrow L^2(\mathbb {R}^2), \qquad (U_n \psi )(\textbf{x}) = e^{i a_n \arg (\textbf{x}- \textbf{x}_n)} \, \psi (\textbf{x}), \end{aligned}$$

for $n \in \{1, \ldots , N\}$. Notice that $U_n \psi$ is indeed a *single-valued* function in $L^2(\mathbb {R}^2)$, given that $e^{2\pi i a_n } = 1$. By composition, we proceed to define the unitary operator $U:= U_1 \cdots \, U_N$. Using the basic identity $\nabla \arg (\textbf{x}- \textbf{x}_n) = \frac{(\textbf{x} - \textbf{x}_{n})^{\perp }}{|\textbf{x} - \textbf{x}_{n}|^2}$, one easily gets

$$\begin{aligned} U \,H_{N}\, U^{-1} = \left( - \,i \nabla + \sum _{n \,=\, 1}^{N}\, (\alpha _n - a_n)\,{(\textbf{x} - \textbf{x}_{n})^{\perp } \over |\textbf{x} - \textbf{x}_{n}|^2} \right) ^{\!2}, \end{aligned}$$

which accounts for the above statement. Let us also point out that the configuration with flux intensities $(\alpha _n)_{n \,\in \, \{1, \ldots , N\}}$ can be mapped to that with opposite parameters $(-\alpha _n)_{n \,\in \, \{1, \ldots , N\}}$ exploiting an obvious conjugation symmetry.

Concerning the fluxes’ distribution, we only assume that their positions are distinct, i.e., that there exists an $$r_{*} $$ fulfilling

$$\begin{aligned} 0< r_{*} <\tfrac{1}{2} \displaystyle \min _{m,n \,\in \, \{1,\,\dots ,N\}} \left|\textbf{x}_{m} - \textbf{x}_{n}\right|. \end{aligned}$$

Accordingly, we can consider a partition of unity given by a family of $C^{2}$ functions $(\xi _{n})_{n \,\in \, \{0,1, \ldots , N\}}: \mathbb {R}^{2} \rightarrow [0,1]$ such that:

1.3 $$\begin{aligned}&\displaystyle \textrm{supp}\,\xi _{n} \subset B_{r_{*}}(\textbf{x}_{n}) \quad \text{ and } \quad \xi _{n}\big |_{B_{r_{*}/2}(\textbf{x}_{n})} \equiv 1\,, \qquad \forall \; n \in \{1,\dots ,N\}\,, \end{aligned}$$

1.4 $$\begin{aligned}&\displaystyle \sum _{n \,=\, 0}^{N} \, \xi _{n}^2 = 1\,, \end{aligned}$$

where we denoted by $B_{\varrho }(\textbf{x})$ the open disk of radius $\varrho > 0$ and center $\textbf{x}$. The above assumptions ensure, in particular, that

1.5 $$\begin{aligned} \textrm{supp}\,\xi _{m} \cap \, \textrm{supp}\,\xi _{n} = \varnothing \,, \qquad \forall \; m \ne n \,; \end{aligned}$$

1.6 $$\begin{aligned} \exists \, R > 0 \quad \hbox { s.t. } \quad \xi _0\big |_{\mathbb {R}^2 \setminus B_{R}(\textbf{0})} = 1 \,. \end{aligned}$$

In view of the above positions, we further introduce the notations, for $n \in \left\{ 1, \dots , N \right\}$,

$$\begin{aligned} \textbf{S}_{n}:= {\sum _{m \,\ne \, n}}\,\textbf{A}_{m}, \qquad \textbf{S}_{0}:= {\sum _{m \,=\, 1}^{N}}\,\textbf{A}_{m}, \end{aligned}$$

to denote the regular part of the magnetic potential in a neighborhood of the *n*-th flux. Furthermore, we set $\check{\textbf{S}}_{n}(\textbf{x}):= \textbf{S}_{n}(\textbf{x}) - \textbf{S}_{n}(\textbf{x}_{n})$, so that

1.7 $$\begin{aligned} \left|\check{\textbf{S}}_{n}(\textbf{x})\right| \leqslant c\,|\textbf{x}-\textbf{x}_n|, \qquad \forall \; n \!\in \!\{1, \dots , N\},\;\, \forall \; \textbf{x} \in \textrm{supp}\,\xi _{n}. \end{aligned}$$

### The Friedrichs Extension

A distinguished self-adjoint realization of the operator (1.2) is the Friedrichs one. In this connection, let us consider the quadratic form associated to the positive operator $H_N$, i.e.,

1.8 $$\begin{aligned} Q_{N}[\psi ]:= \left\| \left( - i \nabla + {\sum _{n \,=\, 1}^{N}} \,\textbf{A}_{n} \right) \psi \right\| _{2}^{\,2}, \end{aligned}$$

which is well defined at least on $C^{\infty }_{\textrm{c}}(\mathbb {R}^2 \,{\setminus }\, \{\textbf{x}_{1},\dots ,\textbf{x}_{N}\})$. Using the natural norm $\Vert \psi \Vert _{N}^2:= \Vert \psi \Vert _{2}^{2} + Q_{N}[\psi ]$, we can identify the quadratic form associated to the Friedrichs extension of ${H}_{N}$, namely,

1.9 $$\begin{aligned} Q_{N}^{(\text {F})}[\psi ] = Q_{N}[\psi ], \qquad \mathscr {D}\big [Q_{N}^{(\text {F})}\big ] = \overline{C^{\infty }_{\text {c}}(\mathbb {R}^2 \setminus \{{\textbf {x}}_{1},\dots ,{\textbf {x}}_{N}\})}^{\;\Vert \,\cdot \,\Vert _{N}}. \end{aligned}$$

For later purposes, let us denote by $\left\langle f \right\rangle _n: (0,+\infty ) \rightarrow \mathbb {C}$ the angular average around $\textbf{x}_{n}$ of any function $f: \mathbb {R}^2 \rightarrow \mathbb {C}$, i.e.,

$$\begin{aligned} \left\langle f \right\rangle _{n}\!(r_n): = \frac{1}{2\pi r_n} \int _{\partial B_{r_n}(\textbf{x}_n)}\hspace{-0.3cm} \textrm{d}\Sigma _n \, f = \frac{1}{2 \pi } \int _0^{2\pi } \textrm{d}\vartheta _n \, f(r_n,\theta _n), \end{aligned}$$

where $r_n: = \left| \textbf{x}- \textbf{x}_n \right|$ and $\vartheta _n:= \textrm{arg}(\textbf{x}- \textbf{x}_n)$ are local polar coordinates around $\textbf{x}_n$ and we have committed a little abuse of notation setting $f(x_n,\vartheta _n):= f(\textbf{x}(x_n,\vartheta _n))$. As we are going to see, exactly as in the single-flux case (see [18, Proposition 1.1]), one of the key properties of the Friedrichs extension is the vanishing of the angular average around each flux of any function $\Psi$ in its domain. Namely, no singular behavior is present in $\Psi$ and the terms $\nabla \Psi$ and $\textbf{A}_n \Psi$ are separately in $L^2(\mathbb {R}^2)$.

#### Proposition 1.2

(Friedrichs extension). Let $N \in \mathbb {N}$ and, for all $n = 1, \ldots , N$, let $\alpha _n \in (0,1)$. Then,

i) The quadratic form $Q_{N}^{(\textrm{F})}$ defined in (1.9) is closed and nonnegative. Its domain is given by
  1.10 $$\begin{aligned} \mathscr {D}\big [Q_{N}^{(\text {F})}\big ] = \left\{ \psi \in H^1(\mathbb {R}^2)\; \big |\;{\textbf {A}}_{n} \psi \in L^2(\mathbb {R}^2),\;\, \forall \; n \!\in \! \left\{ 1,\dots ,N \right\} \right\} . \end{aligned}$$
  Moreover, for any $\psi \in \mathscr {D}\big [Q_{N}^{(\textrm{F})}\big ]$ and for all $n \in \{1,\dots ,N\}$,
  1.11 $$\begin{aligned} \lim _{r_n \rightarrow \, 0^+} \left\langle |\psi |^2 \right\rangle _{\!n} = 0 , \qquad \lim _{r_n \rightarrow \, 0^+} r_n^2 \left\langle |\partial _{r_n}\psi |^2 \right\rangle _{\!n} = 0. \end{aligned}$$
ii) The self-adjoint operator $H_{N}^{(\textrm{F})}$ associated to $Q_{N}^{(\textrm{F})}$ acts as $H_{N}$ on the domain
  1.12 $$\begin{aligned} \mathscr {D}\big (H_{N}^{(\text {F})}\big ) = \left\{ \psi \in \mathscr {D}\big [ Q_{N}^{(\text {F})} \big ] \;\Big |\; H_{N}\psi \in L^2(\mathbb {R}^2) \right\} . \end{aligned}$$

### Derivation of the Perturbed Quadratic Forms

Of course, there are different available approaches to classify all the self-adjoint extensions of a given symmetric operator. For Schrödinger operators with singular magnetic and/or electric potentials and without direct access to the deficiency spaces, however, one must guess either the expression of the quadratic forms identifying the extensions or the local admissible singularities of the functions in the domain of the latter. In both cases, the fact that the so-obtained self-adjoint operators exhaust all the possible realizations is usually proven a posteriori. This means that in either ways an initial guess is unavoidable. However, the boundary conditions labeling the extensions so identified provide the expression of the trace operator which allows to apply directly Kreǐn’s theory and thus complete the solution of the problem. Note, however, that the tails of the AB vector potentials make the boundary conditions inherited from Kreǐn’s theory not so transparent and the corresponding natural parametrization not convenient in general because one cannot write explicitly the asymptotic behavior of the wave functions close to the fluxes (see Proposition 2.11 and the discussion thereafter).

Since we value the explicit characterization of the local behavior, in this work we adopt the quadratic form approach. We start by presenting a derivation of the quadratic forms corresponding to self-adjoint extensions. As a matter of fact, there is a natural parametrization of these forms in terms of Hermitian matrices on the space $\mathbb {C}^N$ (see (1.17) and (1.21)), representing the coefficients of the local singularities in the wave functions. Equivalently, following the alternative approach based on the classification of the admissible local singularities, one is led to the very same natural parametrization (see Remark 1.6). Note, however, that it is hard to figure out *a priori* the regular terms in the local asymptotic expansions. This difficulty is due to the non-local effect generated by the long-range magnetic potentials associated to the other fluxes.

To derive the explicit expressions of the quadratic forms extending (1.9), one can perform the computation described hereafter (see [18, Eqs. (1.24)-(1.27)] for a comparison with the single-flux case). The key idea is that, as for a single flux, the regular wave functions in the Friedrichs realization’s domain can be perturbed by adding defect functions with prescribed singularities at the fluxes.

For $n \in \{1,\dots ,N\}$ and $\lambda > 0$, let us then consider the two defect functions $G^{(\ell _n)}_{\lambda ,n} \in L^2(\mathbb {R}^2)$, $\ell _n \in \{0,-1\}$, associated to the configuration with a single AB flux of intensity $\alpha _n \in (0,1)$, placed at the point $\textbf{x}_n \in \mathbb {R}^2$. Here, $\ell _n$ singles out the angular momentum subspace (*s* and *p* *-waves* only). The functions $G^{(\ell _n)}_{\lambda ,n}$ are identified as the unique solutions of the deficiency equation

1.13 $$\begin{aligned} \left((- i \nabla + \textbf{A}_{n})^{2} + \lambda ^2\right) G^{(\ell _n)}_{\lambda ,n} = 0, \qquad \hbox { in } \; \mathbb {R}^2 {\setminus } \{\textbf{x}_{n}\}, \end{aligned}$$

i.e., explicitly,

1.14 $$\begin{aligned} G^{(\ell _n)}_{\lambda ,n}(r_n,\theta _n) = \lambda ^{|\ell _n + \alpha _n|}\, K_{|\ell _n + \alpha _n|}(\lambda \,r_n)\, \frac{e^{i\, \ell _n \theta _n}}{\sqrt{2\pi }}\,,\qquad \hbox {for }\, \ell _n \in \{0,-1\}\,,\nonumber \\ \end{aligned}$$

where $K_{\nu }$ is the modified Bessel function of second kind, *a.k.a.* Macdonald function. Let us stress that $G^{(\ell _n)}_{\lambda ,n}$ is square-integrable [40, Eq. 6.521.3] with $L^2(\mathbb {R}^2)$ norm

$$\begin{aligned} \left\Vert G^{(\ell _n)}_{\lambda ,n}\right\Vert_{2}^{2} = {\pi \, |\ell _n + \alpha _n| \over 2\sin (\pi \,\alpha _n)}\; \lambda ^{2\,|\ell _n + \alpha _n|-2} , \end{aligned}$$

and has the following asymptotics in a neighborhood of $\textbf{x}_n$:1.15Notice the singular term $\sim \, r_n^{-|\ell _n + \alpha _n|}$ with coefficient independent of $\lambda$, which ensures that $G^{(\ell _n)}_{\lambda ,n} \notin \mathscr {D}\big [Q_{N}^{(\textrm{F})}\big ]$ since it cannot fulfill the asymptotic conditions in (1.11).

In order to set the singular behavior around $\textbf{x}_n$ of the wave functions, we perturb functions $\phi _{\lambda } \in \mathscr {D}\big [Q_{N}^{(\textrm{F})}\big ]$ by adding a term of the form

$$\begin{aligned} \left( \chi _{\lambda } \textbf{q} \right)(\textbf{x}):= \sum _{n \,=\, 1}^{N} \, e^{-i \textbf{S}_{n}(\textbf{x}_n) \cdot (\textbf{x} - \textbf{x}_n)}\, \xi _{n}(\textbf{x})\, \sum _{\ell _n \,\in \, \{0,-1\}} \, q^{(\ell _n)}_{n}\, G^{(\ell _n)}_{\lambda ,n}(\textbf{x}-\textbf{x}_n), \end{aligned}$$

where $\textbf{q} = \big (q_1^{(0)}, q_1^{(-1)}, q_2^{(0)},\, \ldots , q_N^{(0)}, q_N^{(-1)}\big ) \in \mathbb {C}^{2N}$ are free coefficients, the functions $\xi _n$, $n = 1, \ldots , N$, belong to a partition of unity as introduced in (1.3) and (1.4), and the phase factor $e^{-i \textbf{S}_{n}(\textbf{x}_n) \cdot (\textbf{x} - \textbf{x}_n)}$ has been inserted for later convenience. Assuming for the sake of simplicity that $\phi _{\lambda } \in C^{\infty }_{\text {c}}(\mathbb {R}^2{\setminus }\{{\textbf {x}}_1,\dots , {\textbf {x}}_{N}\})$ and formally evaluating the expectation of $H_N$ on $\psi = \phi _{\lambda } + \chi _{\lambda } \textbf{q}$, we get

1.16 $$\begin{aligned}&\left\langle \psi \left|H_{N}^{(\textrm{F})}\right|\psi \right\rangle = \left\langle \phi _{\lambda }\left|H_{N}^{(\textrm{F})}\right|\phi _{\lambda }\right\rangle \nonumber \\&\quad + 2 \textstyle \sum \limits _{n,\ell _n} \Re \left[ q^{(\ell _n)}_{n} \left\langle \phi _{\lambda }\left|H_{N}^{(\textrm{F})}\right|e^{-i \textbf{S}_{n} (\textbf{x}_n) \cdot (\textbf{x} - \textbf{x}_n)}\, \xi _{n} G^{(\ell _n)}_{\lambda ,n}\right\rangle \right]\nonumber \\&\quad +\! \!\!\textstyle \sum \limits _{n,\ell _n,\ell _n^{\prime }} \!\!\big ({q^{(\ell _n)}_{n}}\big )^* q^{(\ell _n^{\prime })}_{n}\! \left\langle e^{-i \textbf{S}_{n}(\textbf{x}_n) \cdot (\textbf{x} - \textbf{x}_n)}\, \xi _{n} G^{(\ell _n)}_{\lambda ,n}\left|H_{N}^{(\textrm{F})}\right|e^{-i \textbf{S}_{n}(\textbf{x}_n) \cdot (\textbf{x} - \textbf{x}_n)}\,\xi _{n} G^{(\ell _n^{\prime })}_{\lambda ,n}\right\rangle \nonumber \\\,. \end{aligned}$$

Note the absence of off-diagonal terms in the last sum, thanks to the disjoint supports of the functions $\xi _n$. For $\textbf{x} \ne \textbf{x}_{n}$ we haveand, since the support of $\phi _{\lambda }$ does not comprises $\textbf{x}_{n}$, we can integrate by parts without getting any boundary term (we shall return on this point in the proof of Theorem 1.4), so obtaining

$$\begin{aligned}&\left\langle \phi _{\lambda }\left|H_{N}^{(\textrm{F})}\right|e^{-i \textbf{S}_{n}(\textbf{x}_n) \cdot (\textbf{x} - \textbf{x}_n)}\,\xi _{n} G^{(\ell _n)}_{\lambda ,n}\right\rangle \\&\quad = 2 \left\langle \left(-i \nabla \!+\! \textbf{A}_{n}\right) \phi _{\lambda }\left|\,e^{-i \textbf{S}_{n}(\textbf{x}_n) \cdot (\textbf{x} - \textbf{x}_n)} \big (\check{\textbf{S}}_{n} \,\xi _{n} - i \nabla \xi _{n} \big ) G^{(\ell _n)}_{\lambda ,n}\right. \right\rangle \\&\qquad + \left\langle \phi _{\lambda }\left|e^{-i \textbf{S}_{n}(\textbf{x}_n) \cdot (\textbf{x} - \textbf{x}_n)}\left( \big (\check{\textbf{S}}_{n}^2 - \lambda ^2\big )\, \xi _{n} + 2 \textbf{S}_n(\textbf{x}_n) \cdot (\check{\textbf{S}}_n \xi _{n} - i \nabla \xi _{n}) + \Delta \xi _{n} \right)\! G^{(\ell _n)}_{\lambda ,n}\right. \right\rangle . \end{aligned}$$

Similarly, we deduce

$$\begin{aligned}&\left\langle e^{-i \textbf{S}_{n}(\textbf{x}_n) \cdot (\textbf{x} - \textbf{x}_n)}\,\xi _{n} G^{(\ell _n)}_{\lambda ,n}\left|H_{N}^{(\textrm{F})}\right|e^{-i \textbf{S}_{n}(\textbf{x}_n) \cdot (\textbf{x} - \textbf{x}_n)}\, \xi _{n} G^{(\ell _n^{\prime })}_{\lambda ,n}\right\rangle \\&\quad = 2 \left\langle \xi _{n} G^{(\ell _n)}_{\lambda ,n}\left| \left( - i \nabla \xi _{n} \right) \!\cdot \! \left(-i \nabla \!+\! \textbf{A}_n\right) G^{(\ell _n^{\prime })}_{\lambda ,n} \right. \right\rangle \\&\qquad + \left\langle \xi _{n} G^{(\ell _n)}_{\lambda ,n}\left| \Big (\big (\check{\textbf{S}}_n^2\! - \lambda ^2\big ) \xi _{n} + 2 \big (\check{\textbf{S}}_n \!\cdot \! \textbf{A}_n\big )\xi _{n} - \Delta \xi _{n} \Big ) G^{(\ell _n^{\prime })}_{\lambda ,n} \right. \right\rangle \\&\qquad + 2 \left\langle \xi _{n} G^{(\ell _n)}_{\lambda ,n}\left| \,\check{\textbf{S}}_n \!\cdot \! (-i \nabla ) \big (\xi _{n} G^{(\ell _n^{\prime })}_{\lambda ,n}\big )\right. \right\rangle , \end{aligned}$$

so that, symmetrizing the last term of (1.16) and integrating by parts, we get

$$\begin{aligned}&\textstyle \sum \limits _{n,\ell _n,\ell _n^{\prime }} \big ({q^{(\ell _n)}_{n}}\big )^* q^{(\ell _n^{\prime })}_{n}\left\langle e^{-i \textbf{S}_{n}(\textbf{x}_n) \cdot (\textbf{x} - \textbf{x}_n)}\,\xi _{n} G^{(\ell _n)}_{\lambda ,n}\left|H_{N}^{(\textrm{F})}\right|e^{-i \textbf{S}_{n}(\textbf{x}_n) \cdot (\textbf{x} - \textbf{x}_n)}\,\xi _{n} G^{(\ell _n^{\prime })}_{\lambda ,n}\right\rangle \\&\quad = \textstyle \sum \limits _{n,\ell _n,\ell _n^{\prime }} \big ({q^{(\ell _n)}_{n}}\big )^* q^{(\ell _n^{\prime })}_{n} \bigg [ \left\langle G^{(\ell _n)}_{\lambda ,n} \left|(\nabla \xi _{n})^2 G^{(\ell _n^{\prime })}_{\lambda ,n}\right. \right\rangle \\&\qquad + \left\langle \xi _{n} G^{(\ell _n)}_{\lambda ,n}\left| \Big (\big (\check{\textbf{S}}_n^2\! - \lambda ^2\big ) \xi _{n} + 2 \big (\check{\textbf{S}}_n \!\cdot \! \textbf{A}_n\big )\xi _{n} \Big ) G^{(\ell _n^{\prime })}_{\lambda ,n} \right. \right\rangle \\&\qquad + 2 \left\langle \xi _{n} G^{(\ell _n)}_{\lambda ,n}\left| \,\check{\textbf{S}}_n \!\cdot \! (-i \nabla ) \big (\xi _{n} G^{(\ell _n^{\prime })}_{\lambda ,n}\big )\right. \right\rangle \bigg ] \,. \end{aligned}$$

Exploiting the identity

$$\begin{aligned} \left\Vert \psi \right\Vert_{2}^2&= \left\Vert \phi _{\lambda } \right\Vert_{2}^2 + 2 \textstyle \sum \limits _{n,\ell _n} \Re \Big [ q^{(\ell _n)}_{n} \left\langle \phi _{\lambda }\left| \,e^{-i \textbf{S}_{n}(\textbf{x}_n) \cdot (\textbf{x} - \textbf{x}_n)}\,\xi _{n} G^{(\ell _n)}_{\lambda ,n}\right. \right\rangle \Big ] \\&\quad + \textstyle \sum \limits _{n,\ell _n,\ell _n^{\prime }} \big ({q^{(\ell _n)}_{n}}\big )^* q^{(\ell _n^{\prime })}_{n} \left\langle \xi _{n} G^{(\ell _n)}_{\lambda ,n}\left|\xi _{n} G^{(\ell _n^{\prime })}_{\lambda ,n}\right. \right\rangle , \end{aligned}$$

we are finally led to consider the expression1.17where1.181.191.20and we have introduced the Hermitian matrix

1.21 $$\begin{aligned} B: = \left(B_{m\,n}^{(\ell _m \ell '_n)}\right)_{m,n \in \{1, \ldots , N\}; \, \ell _m, \ell '_n \in \{0,-1\}} \in M_{2N,\,\textrm{Herm}}(\mathbb {C}), \end{aligned}$$

labeling the form. Notice that, thanks to the properties of the cutoff functions $\xi _n$ and (1.7), we have $\zeta _{1,n}, \zeta _{2,n}, \check{\textbf{S}}_n \cdot \textbf{A}_n \xi _n \in L^{\infty }(\mathbb {R}^2)$. Therefore, all the scalar products appearing in the quadratic form are well posed: in particular, for the last term in $\Xi ^{(\ell _n \ell _n^{\prime })}_{n}(\lambda )$, one has to use that $\check{\textbf{S}}_n$ linearly vanishes around $\textbf{x}_n$ to compensate the extra singularity due to the gradient. Since it can be checked by direct inspection that $\Xi ^{(\ell _n \ell _n^{\prime })}_{n}(\lambda ) = \big ({\Xi ^{(\ell _n^{\prime } \ell _n)}_{n}(\lambda )}\big )^{*}$ and *B* is Hermitian, we also infer that the form is real.

### Main Results

Our first result is about the quadratic forms $Q^{(B)}_{N}$ defined in (1.17) on their natural domain of definition, i.e.,

1.22 $$\begin{aligned} \mathscr {D}\big [Q^{(B)}_{N}\big ]&{:=} \bigg \{\psi \!\in \! L^2(\mathbb {R}^2)\,\bigg |\, \psi = \phi _{\lambda } {+} \sum _{n \,=\, 1}^{N}\, e^{-i {\textbf {S}}_{n}({\textbf {x}}_n) \cdot ({\textbf {x}} - {\textbf {x}}_n)}\, \xi _{n}\, \sum _{\ell _n \,\in \, \{0,-1\}}\, q^{(\ell _n)}_{n}\, G^{(\ell _n)}_{\lambda ,n} \,,\, \nonumber \\ {}&\quad \phi _{\lambda } \!\in \! \mathscr {D}\big [Q_{N}^{(\text {F})}\big ],\;\, q^{(\ell _n)}_{n} \!\in \! \mathbb {C},\;\, n = 1,\dots , N,\;\, \ell _n = 0,-1 \bigg \} \,. \end{aligned}$$

#### Theorem 1.3

(Quadratic forms. $Q^{(B)}_{N}$). Let $N \in \mathbb {N}$ and, for all $n = 1,\dots ,N$, let $\alpha _n \in (0,1)$ and $\xi _{n}: \mathbb {R}^2 \rightarrow [0,1]$ fulfill (1.3)–(1.6). Then, for any Hermitian matrix $B \in M_{2N,\,\textrm{Herm}}(\mathbb {C})$,

i) the quadratic form $Q^{(B)}_{N}$ defined by (1.17) is well posed on the domain (1.22); moreover, it is independent of $\lambda > 0$ and of the choice of $(\xi _{n})_{n \,=\, 1,\,\dots ,N}$;
ii) $Q^{(B)}_{N}$ is also closed and bounded from below on the same domain.

Next we show that the self-adjoint operators associated to the forms $Q^{(B)}_{N}$ identify all self-adjoint realizations of $H_N$. Besides the derivation of the domain and explicit action of such operators, including the boundary conditions satisfied by the functions contained therein, the major content of the result reported below is the fact that *all* the self-adjoint extensions are contained in the family. This is obtained indirectly through an alternative parametrization of the family via Kreǐn’s theory (see also the subsequent Remark 1.7).

#### Theorem 1.4

(Self-adjoint extensions $$ H_{N}^{(B)}$$). Under the same assumptions of Theorem 1.3, for any Hermitian matrix $$ B \in M_{2N,\,\textrm{Herm}}(\mathbb {C})$$ the operator $$ H_{N}^{(B)} $$ associated to the quadratic form $$Q^{(B)}_{N}$$ has domain1.23where $$ L_{\lambda }: = \left(\tfrac{\pi \,\lambda ^{2|\ell '_n + \alpha _n|}}{ 2\sin (\pi \alpha _n)}\,\delta _{mn}\,\delta _{\ell _m \ell _n^{\prime }} \right)_{m,n \in \{1, \ldots , N\}; \, \ell _m, \ell '_n \in \{0,-1\}} \in M_{2N,\,\textrm{Herm}}(\mathbb {C})$$, and action

$$\begin{aligned}&\left( H_{N}^{(B)} \!+\! \lambda ^2\right)\! \psi = \left( H_{N}^{(\textrm{F})} \!+\! \lambda ^2\right)\! \phi _{\lambda }\\&\quad + \sum _{n,\ell _n} q^{(\ell _n)}_{n}\, e^{-i \textbf{S}_{n}(\textbf{x}_n) \cdot (\textbf{x} - \textbf{x}_n)} \Big [ 2\big (\check{\textbf{S}}_{n}\xi _{n}\! - i \nabla \xi _{n} \big ) \left(-i \nabla \!+\! \textbf{A}_{n}\right)G^{(\ell _n)}_{\lambda ,n} \\&\quad + \left( \check{\textbf{S}}_{n}^2 \xi _{n}\! + 2\check{\textbf{S}}_{n} \!\cdot \! (-i \nabla \xi _{n}) - \Delta \xi _{n} \right)\! G^{(\ell _n)}_{\lambda ,n} \Big ]\,. \end{aligned}$$

Moreover, the $(2N)^2$-real-parameters family $H_{N}^{(B)}$, B $\in M_{2N,\,\text {Herm}}(\mathbb {C}) \cup \left\{ \infty \right\}$, exhausts all possible self-adjoint realizations of the operator ${H}_{N}$.

#### Remark 1.5

(Friedrichs extension). The Friedrichs Hamiltonian is formally recovered for “$B = \infty$” and from now on we may use the notation $B = \infty$ to identify the Friedrichs extension. Indeed, when all components of *B* diverge, all the charge parameters $q_{n}^{(\ell _n)}$ are set equal to zero. In this case, the boundary conditions in (1.23) for $n \in \{1,\dots ,N\}$, $\ell _n \in \{0,-1\}$, read

$$\begin{aligned} \left\langle \Big ( |\ell _n + \alpha _n|\, \phi _{\lambda } + r\, \partial _r \phi _{\lambda }\Big ) \tfrac{e^{-i\, \ell _n \theta }}{\sqrt{2\pi }} \right\rangle _n = \mathcal {O}\left(r_n^{|\ell _n + \alpha _n|}\right), \qquad \hbox { for }\, r_n \rightarrow 0^{+}. \end{aligned}$$

This asymptotic behavior is apparently missing in the characterization of the Friedrichs domain (1.10), but it is in fact encoded in the requirement $H_{N} \phi _\lambda \in L^2(\mathbb {R}^2)$ (see also [21, §3.1]). In fact, by decomposition in angular harmonics centered at $\textbf{x}_n$ and a finer analysis of the associated radial problems, building on the results presented in [29] it can be shown that, in the limit $\textbf{x}\rightarrow \textbf{x}_n$,

1.24 $$\begin{aligned} \phi _{\lambda }(\textbf{x}) = c_n^{(0)}\,|\textbf{x}- \textbf{x}_n|^{\alpha } + c_n^{(-1)}\,|\textbf{x}- \textbf{x}_n|^{1-\alpha } \,e^{-i \arg (\textbf{x}- \textbf{x}_n)} + o\big (|\textbf{x}- \textbf{x}_n|\big ),\nonumber \\ \end{aligned}$$

for some suitable mutually independent coefficients $c_n^{(0)},c_n^{(-1)} \in \mathbb {C}$.

#### Remark 1.6

(Local singularities) For a generic $B \in M_{2N,\,\textrm{Herm}}(\mathbb {C})$, the boundary conditions in (1.23) fulfilled by any $\psi \in \mathscr {D}\big (H_{N}^{(B)}\big )$ can be equivalently written as follows, using the asymptotic expansion of $G_{\lambda ,n}^{(\ell _n)}$ (see (1.15)) and that of any element $\phi _{\lambda } \in \mathscr {D}\big (H_{N}^{(\textrm{F})}\big )$ (see (1.24)), for any $n \in \{1,\ldots ,N\}$:

1.25 $$\begin{aligned} \psi ({\textbf {x}})&= {d^{(0)}_{n} \over |{\textbf {x}}- {\textbf {x}}_n|^{\alpha _n}}+ c_n^{(0)}\, |{\textbf {x}}- {\textbf {x}}_n|^{\alpha _n}\nonumber \\ {}&\quad + \left( {d^{(-1)}_{n} \over |{\textbf {x}}- {\textbf {x}}_n|^{1 - \alpha _n}} + c_n^{(-1)} |{\textbf {x}}- {\textbf {x}}_n|^{1 - \alpha _n} \right) e^{-i\, \arg ({\textbf {x}}- {\textbf {x}}_n)}\nonumber \\ {}&\quad - i\, d^{(0)}_{n}\,\frac{{\textbf {S}}_{n}({\textbf {x}}_n) \cdot ({\textbf {x}} - {\textbf {x}}_n) }{|{\textbf {x}}- {\textbf {x}}_n|^{\alpha _n}}-i\,d^{(-1)}_{n}\, \frac{{\textbf {S}}_{n}({\textbf {x}}_n) \cdot ({\textbf {x}} - {\textbf {x}}_n) }{|{\textbf {x}}- {\textbf {x}}_n|^{1 - \alpha _n}}\, e^{-i\, \arg ({\textbf {x}}- {\textbf {x}}_n)} \nonumber \\ {}&\quad + o\big (|{\textbf {x}}- {\textbf {x}}_n|\big )\,, \end{aligned}$$

where now the coefficients $c_n^{(\ell _n)},d_n^{(\ell _n)} \in \mathbb {C}$ ($\ell _n = 0,-1$) are linked by the following linear relation

$$\begin{aligned} c_n^{(0)}&= \tfrac{1}{2^{\alpha _n}\,\Gamma (\alpha _n+1)}\, \sum _{m \,=\, 1}^{N} \left[\tfrac{2^{1-\alpha _m}}{\Gamma (\alpha _m)}\, B_{nm}^{(0,0)} d_m^{(0)} + \tfrac{2^{\alpha _m}}{\Gamma (1-\alpha _m)}\, B_{nm}^{(0,-1)} d_m^{(-1)}\right], \\ c_n^{(-1)}&= \tfrac{1}{2^{1-\alpha _n}\Gamma (2 - \alpha _n)}\, \sum _{m \,=\, 1}^{N} \left[ \tfrac{2^{1-\alpha _m}}{\Gamma (\alpha _m)}\, B_{nm}^{(-1,0)} d_m^{(0)} + \tfrac{2^{\alpha _m}}{\Gamma (1-\alpha _m)}\, B_{nm}^{(-1,-1)} d_m^{(-1)} \right]. \end{aligned}$$

Note that the two terms in (1.25) depending on $\textbf{S}_{n}(\textbf{x}_n)$, i.e., the phase shifts generated in $\textbf{x}_n$ by the other fluxes, contain spherical harmonics with angular momentum $\ell _n$ ranging from $-2$ to $+1$: this can be easily checked using the identity

1.26 $$\begin{aligned} {\textbf {S}}_{n}({\textbf {x}}_n) \cdot ({\textbf {x}}\! - \!{\textbf {x}}_n) \!=\! \left( \tfrac{S_{n,x} - i S_{n,y}}{2}\,e^{i \arg ({\textbf {x}}- {\textbf {x}}_n)} \!+\! \tfrac{S_{n,x} + i S_{n,y}}{2}\,e^{-i \arg ({\textbf {x}}- {\textbf {x}}_n)}\right) |{\textbf {x}}-{\textbf {x}}_n|\,. \end{aligned}$$

Hence, the local radial behavior of such terms is $|\textbf{x}- \textbf{x}_n|^{\alpha _n}$ and $|\textbf{x}- \textbf{x}_n|^{1-\alpha _n}$, respectively. In particular, this implies that the domain of a generic self-adjoint extension is obtained by modifying the Friedrichs domain in the *d*-wave subspace, on top of adding singular terms in the *s*- and *p*-wave sectors. Furthermore, unlike for the Friedrichs extension, in the domain of any other self-adjoint realization, *both* the regular terms $|\textbf{x}- \textbf{x}_n|^{\alpha _n}$ and $|\textbf{x}- \textbf{x}_n|^{1-\alpha _n}$ are present in the asymptotic expansion in *each* angular sector $\ell _n = 0$ and $\ell _n = -1$.

#### Remark 1.7

(Kreǐn representation). As anticipated, a key point in the proof of Theorem 1.4 is an alternative representation of the self-adjoint realizations of $$ H_N $$ via a straightforward application of Kreǐn theory. More precisely, one can show (see Proposition 2.10) that all self-adjoint extensions of $$ H_N$$ can be written in the following form, for $$ z \in \mathbb {C} {\setminus } \mathbb {R} $$ and $$ \Theta \in M_{2N,\,\text {Herm}}(\mathbb {C}) \cup \left\{ \infty \right\} $$:

1.27 $$\begin{aligned} \mathscr {D}\big (H_{N}^{(\Theta )}\big )= & {} \Big \{ \psi \in L^2(\mathbb {R}^2)\;\Big |\; \psi = \varphi _{z} + \mathcal {G}(z) \textbf{q},\; \varphi _{z} \in \mathscr {D}\big (H_{N}^{(\textrm{F})}\big ),\nonumber \\{} & {} \quad \textbf{q} \!\in \mathbb {C}^{2N}, \; \varvec{\tau } \varphi _z = \big [\Theta + \Lambda (z)\big ] \textbf{q} \Big \}, \nonumber \\{} & {} \quad \big (H_{N}^{(\Theta )} - z\big ) \psi = \big (H_{N}^{(\textrm{F})} - z\big ) \varphi _z. \end{aligned}$$

Here, $\varvec{\tau }$ stands for the trace map $\varvec{\tau }:= \bigoplus _{n = 1, \ldots , N; \ell _n \in \{0,-1\}} \tau _{n}^{(\ell _n)}: \mathscr {D}\big (H_{N}^{(\textrm{F})}\big ) \rightarrow \mathbb {C}^{2N}$, where (*cf.* (1.23))

1.28 $$\begin{aligned} \tau _{n}^{(\ell _n)} \psi := \lim _{r \rightarrow 0^{+}} \tfrac{\pi \,2^{|\ell _n + \alpha _n|} \,\Gamma \big (|\ell _n + \alpha _n|\big )}{r^{|\ell _n + \alpha _n|}}\,\left\langle \Big ( |\ell _n + \alpha _n|\,\psi + r\,\partial _{r} \psi \Big ) \tfrac{e^{-i\, \ell _n \theta }}{\sqrt{2\pi }} \right\rangle _{n}. \end{aligned}$$

The associated single-layer operator is

$$\begin{aligned} \mathcal {G}(z):= \big (\breve{\mathcal {G}}(\bar{z}) \big )^{*}: \mathbb {C}^{2N} \rightarrow L^2(\mathbb {R}^2), \end{aligned}$$

with $\breve{\mathcal {G}}(z):= \varvec{\tau } \, ( H^{(\textrm{F})}_N - z )^{-1}: L^2(\mathbb {R}^2) \rightarrow \mathbb {C}^{2N}$. Moreover, fixing arbitrarily $z_0 \in \mathbb {C} \setminus [0,+\infty )$, we have set

1.29 $$\begin{aligned} \Lambda (z):= \varvec{\tau } \left( \tfrac{1}{2} \left( \mathcal {G}(z_0) + \mathcal {G}(\bar{z}_0) \right) - \mathcal {G}(z) \right): \mathbb {C}^{2N} \rightarrow \mathbb {C}^{2N}. \end{aligned}$$

The two representations of the self-adjoint extensions are completely equivalent, i.e., there is a one-to-one correspondence between the two families, which we denote by $\Theta (B)$, $\Theta : M_{2N,\,\textrm{Herm}}(\mathbb {C}) \mapsto M_{2N,\,\textrm{Herm}}(\mathbb {C})$ (see Proposition 2.11 for the explicit form of such a change of parametrization).

We now focus on the main spectral and scattering properties of the operators $H_{N}^{(B)}$. The crucial ingredient in this framework is an explicit expression of the resolvent operator of any self-adjoint realization. We start by observing that the resolvent of the Friedrichs extension admits a convenient representation (see Proposition 2.1):

1.30 $$\begin{aligned} R^{(\text {F})}_{N}(z){} & {} : = \big ( H^{(\text {F})}_N - z \big )^{-1}\nonumber \\ {}{} & {} = {} \sum _{n \,=\, 0}^{N}\, e^{- i {\textbf {S}}_{n}({\textbf {x}}_n) \cdot ({\textbf {x}} - {\textbf {x}}_n)}\xi _{n}\, R^{(\text {F})}_{n}(z)\, \xi _{n}\,e^{i {\textbf {S}}_{n}({\textbf {x}}_n) \cdot ({\textbf {x}} - {\textbf {x}}_n)}\, \big [1 + T_N(z)\big ]^{-1},\nonumber \\ \end{aligned}$$

where, for any $n \in \{1,\dots ,N\}$, $R^{(\textrm{F})}_{n}(z): = ( H^{(\textrm{F})}_n - z )^{-1}$ and $H^{(\textrm{F})}_n = (-i \nabla \!+\! \textbf{A}_{n})^2$ stands for the Friedrichs realization of the Schrödinger operator corresponding to a single AB flux of intensity $\alpha _n$, placed at $\textbf{x}_{n}$. We recall that the integral kernel of $R^{(\textrm{F})}_{n}(z)$ can be expressed as (see [4, Eq. (3.2)] and [61, Eqs. 10.27.6-7])

1.31 $$\begin{aligned}{} & {} R^{(\textrm{F})}_{n}\big (z; r_n,\theta _n; r'_n,\theta '_n\big )\nonumber \\{} & {} \quad = \sum _{\ell \,\in \, \mathbb {Z}} \tfrac{1}{2 \pi } \; I_{|\ell + \alpha |}\big (\!- i \sqrt{z} \, (r_n \wedge r'_n)\big )\,K_{|\ell + \alpha |}\big (\!- i \sqrt{z} \,(r_n \vee r'_n)\big ) \; e^{i \ell (\theta _n - \theta '_n)} .\nonumber \\ \end{aligned}$$

Here and in the sequel for any complex number $z \in \mathbb {C} {\setminus } \mathbb {R}^{+}$, we always consider the determination of the square root with $\Im \sqrt{z} > 0$. By convention, we identify $R_{0}^{(F)}(z) \equiv R_{0}(z):= (-\Delta - z)^{-1}$ with the resolvent of the free Hamiltonian, whose integral kernel is (see [61, Eqs. 10.27.6-7])

$$\begin{aligned} R_{0}(z; \textbf{x}; \textbf{x}') = \tfrac{i}{4}\, H^{(1)}_{0}\big (\sqrt{z} \;|\textbf{x} - \textbf{x}'|\,\big ) = \tfrac{1}{2 \pi }\, K_{0}\big (\!-i \sqrt{z} \,|\textbf{x} - \textbf{x}'|\big ) . \end{aligned}$$

On the other hand, the operator $T_N$ appearing in (1.30) is given by

1.32 $$\begin{aligned} T_N(z)&:= \sum _{n \,=\, 0}^{N} e^{-i \textbf{S}_{n}(\textbf{x}_n) \cdot (\textbf{x} - \textbf{x}_n)} P_n\, R^{(\textrm{F})}_{n}(z)\, \xi _{n}\,e^{i \textbf{S}_{n}(\textbf{x}_n) \cdot (\textbf{x} - \textbf{x}_n)}\,, \end{aligned}$$

1.33 $$\begin{aligned} P_n&:= 2 \,\left(\check{\textbf{S}}_{n} \xi _{n} - i \nabla \xi _{n}\right) \!\cdot \! (-i\nabla \!+\! \textbf{A}_{n}) + \check{\textbf{S}}_{n}^2 \xi _{n} + 2 \,\check{\textbf{S}}_{n}\! \cdot ( - i \nabla \xi _{n})\! - \Delta \xi _{n}\,. \end{aligned}$$

Combining the above representation of the Friedrichs resolvent with the Kreǐn formula for the resolvent of the self-adjoint extensions (recall Remark 1.7), i.e.,

$$\begin{aligned} R_N^{(B)}(z):= \big ( H^{(\Theta (B))}_N - z \big )^{-1} = R_N^{(\textrm{F})}(z) + \mathcal {G}(z) \big [ \Theta (B) + \Lambda (z) \big ]^{-1} \breve{\mathcal {G}}(z), \end{aligned}$$

where the explicit form of the map $\Theta (B)$ is given in (2.22), we are able to prove the following results.

#### Proposition 1.8

(Spectral properties). Let $N \in \mathbb {N}$ and, for all $n = 1,\dots ,N$, let $\alpha _n \in (0,1)$. Then, for any Hermitian matrix $B \in M_{2N,\,\textrm{Herm}}(\mathbb {C})$,

$$\begin{aligned} \sigma _{\textrm{ac}}\big (H_{N}^{(B)}\big ) = \sigma _{\textrm{ac}}(-\Delta ) = [0,+\infty ). \end{aligned}$$

Furthermore, $\sigma _{\text {disc}}\big (H_{N}^{(\text {F})}\big ) = \varnothing$, while $\sigma _{\text {disc}}\big (H_{N}^{(B)}\big )$ contains at most 2*N* negative eigenvalues. More precisely,

$$\begin{aligned} -\lambda ^2 \in \sigma _{\textrm{disc}}\big (H_{N}^{(B)}\big ) \quad (\lambda > 0) \quad \Longleftrightarrow \quad \ker \left[ \Theta (B) + \Lambda (-\lambda ^2) \right] \ne \varnothing , \end{aligned}$$

and the associated eigenvectors are of the form $\mathcal {G}(-\lambda ^2)\,\textbf{q}$, with $\textbf{q} \in \ker \left[ \Theta (B) + \Lambda (-\lambda ^2) \right]$.

Concerning the scattering, we consider the pair $\big ( H_{N}^{(B)}, - \Delta \big )$ and define the wave operators

$$\begin{aligned} \Omega _{\pm }\left( H_{N}^{(B)}, - \Delta \right) :=\ \textrm{s}\!-\!\lim \limits _{t \rightarrow \mp \infty }\, e^{i t H_{N}^{(B)}} e^{i t \Delta }. \end{aligned}$$

To avoid misunderstandings, let us specify the meaning of completeness for wave operators understood here. Following [69, §XI.3], for any pair of self-adjoint operators *A*, *B* acting in a given Hibert space $\mathcal {H}$, we say that the wave operators $\Omega _{\pm }(A,B)$ are *complete* if

$$\begin{aligned} \hbox {ran}\,\Omega _{\pm }(A,B) = \text{ ran } \,\Omega _{\pm }(B,A) = \hbox {ran}\,P_{\textrm{ac}}(A) , \end{aligned}$$

where $P_{\textrm{ac}}(A)$ stands for the spectral projector onto the absolute continuity subspace of *A*. We recall that whenever the wave operators $\Omega _{\pm }(A,B)$ exist, they are complete if and only if $\Omega _{\pm }(B,A)$ exist as well (see [69, Proposition 3 (vol. III, p. 19)]). On the other hand, *asymptotic completeness* requires also that $\sigma _{\textrm{sc}}(A) = \varnothing$.

#### Proposition 1.9

(Scattering properties). Let $N \in \mathbb {N}$ and, for all $n = 1,\dots ,N$, let $\alpha _n \in (0,1)$. Then, for any $B \in M_{2N,\,\text {Herm}}(\mathbb {C}) \cup \left\{ \infty \right\}$, the wave operators $\Omega _{\pm }(H_{N}^{(B)}, - \Delta )$ exist and are complete.

## Proofs

### The Friedrichs Extension

We discuss first the properties of the Friedrichs extension, which will play a key role in the analysis of all self-adjoint realizations.

#### Proof of Proposition 1.2

*i)* Nonnegativity and closedness are obvious consequences of (1.8) and (1.9), respectively. Let us account for (1.10), showing the reciprocal inclusion of the sets on its left and right sides. On the one hand, we have

$$\begin{aligned} Q_{N}[\psi ] \leqslant 2\, \Vert \nabla \psi \Vert _{2}^{2} + 2 \left\| {\sum _{n \,=\, 1}^{N}}\textbf{A}_{n} \psi \right\| _{2}^{2} \leqslant 2\, \Vert \nabla \psi \Vert _{2}^{2} + 2N\,{\sum _{n \,=\, 1}^{N}} \Vert \textbf{A}_{n} \psi \Vert _{2}^{2}, \end{aligned}$$

which suffices to infer that the r.h.s. of (1.10) is a subset of $\mathscr {D}\big [Q_{N}^{(\textrm{F})}\big ]$. On the other hand, let us consider a partition of unity as in (1.3) and (1.4): we notice that $\textbf{S}_n \xi _{n} \in L^{\infty }(\mathbb {R}^2)$ for all $n \in \{0,1,\dots ,N\}$. Then, starting again from (1.8) and using a variant of the IMS localization formula (see, e.g., [22, Thm. 3.2]), we derive the following chain of inequalities, for any $\epsilon \in (0,1)$ and some suitable $C_\epsilon > 0$:

$$\begin{aligned} Q_{N}[\psi ]&= \sum \limits _{n \,=\, 0}^{N} \left\Vert \left(-i \nabla \!+\! \sum \limits _{m \,=\, 1}^{N} \,\textbf{A}_{m}\right)(\xi _{n} \psi ) \right\Vert_{2}^{2} - \sum \limits _{n \,=\, 0}^{N} \left\Vert \left(\nabla \xi _{n} \right)\psi \right\Vert_{2}^{2} \\&\geqslant (1-\epsilon ) \, \sum \limits _{n \,=\, 0}^{N} \left\Vert \left(-i \nabla \!+\! \textbf{A}_{n}\right)(\xi _{n} \psi ) \right\Vert_{2}^{2} - \tfrac{1- \epsilon }{\epsilon }\, \sum \limits _{n \,=\, 0}^{N} \left\Vert \textbf{S}_{n} \xi _{n} \psi \right\Vert_{2}^{2}\\&\quad - \sum \limits _{n \,=\, 0}^{N} \left\Vert \left(\nabla \xi _{n} \right)\psi \right\Vert_{2}^{2} \\&\geqslant (1-\epsilon )^2 \left\Vert\nabla (\xi _{0} \psi ) \right\Vert_{2}^{2} + (1-\epsilon )^2 \,{\sum \limits _{n \,=\, 1}^{N}} (1-\alpha _n)^2 \left\Vert\nabla (\xi _{n} \psi ) \right\Vert_{2}^{2} \\&\quad + \epsilon (1-\epsilon ) \, {\sum \limits _{n \,=\, 1}^{N}} \min \left\{ 1 \,, \tfrac{(1 - \alpha _n)^2}{\alpha _n^2}\right\} \left\Vert \textbf{A}_n \xi _n\psi \right\Vert_{2}^{2} - C_{\epsilon } \left\Vert \psi \right\Vert_{2}^{2} \\&\geqslant (1-\epsilon )^2 \min _{n\,=\, 1,\dots ,N} (1-\alpha _n)^2 \left\Vert\nabla \psi \right\Vert_{2}^{2} \\&\quad + \epsilon (1-\epsilon ) \left( \min _{n\,=\,1,\dots ,N} \left\{ 1 \,, \tfrac{(1 - \alpha _n)^2}{\alpha _n^2}\right\} \right) {\sum \limits _{n \,=\, 1}^{N}} \left\Vert \textbf{A}_n \xi _n \psi \right\Vert_{2}^{2} - C_{\epsilon } \left\Vert \psi \right\Vert_{2}^{2} \,. \end{aligned}$$

Here, we have used the lower bounds (see [18, Eq. (2.4)])

$$\begin{aligned}{} & {} \left\Vert \left(-i \nabla + \textbf{A}_{n}\right) \psi \right\Vert_{2}^{2} \geqslant (1-\alpha _n)^2 \,\left\Vert\nabla \psi \right\Vert_{2}^{2},\\{} & {} \qquad \left\Vert \left(-i \nabla + \textbf{A}_{n}\right) \psi \right\Vert_{2}^{2} \geqslant \min \left\{ 1 , \tfrac{(1 - \alpha _n)^2}{\alpha _n^2}\right\} \,\left\Vert \textbf{A}_n \psi \right\Vert_{2}^{2}. \end{aligned}$$

Summing up, we infer that for any $c > 0$ small enough, there exists a finite $\gamma _c > 0$ such that

$$\begin{aligned} Q_{\alpha ,N}[\psi ] + \gamma _c\,\Vert \psi \Vert _{2}^{2} \geqslant c \left( \left\Vert\nabla \psi \right\Vert_{2}^{2} + {\sum _{n \,=\, 1}^{N}} \left\Vert \textbf{A}_n\, \xi _n \psi \right\Vert_{2}^{2} \right) . \end{aligned}$$

This shows that $\mathscr {D}\big [ Q_{N}^{(\textrm{F})} \big ]$ is a subset of the r.h.s. of (1.10), thus proving the identity (1.10).

Finally, for any $n \in \{1,\dots ,N\}$ the limits in (1.11) can be deduced by the same arguments described in [18, §2.1], making reference to the representation in polar coordinates centered at $\textbf{x}_{n}$ and building on the square-integrability of $\nabla \psi ,\textbf{A}_{n} \psi$ near $\textbf{x}_{n}$ for any $\psi \in \mathscr {D}\big [ Q_{N}^{(\textrm{F})} \big ]$.

*ii)* Given the sesquilinear form $Q_{N}^{(\textrm{F})}[\psi _1,\psi _2]$ defined by polarization starting from the quadratic form $Q_{N}^{(\textrm{F})}$, the unique operator associated to it (see, e.g., [69, Thm. VIII.15]) iswith $H_{N}^{(\textrm{F})} \psi _{2}:= w$ for all $\psi _{2} \!\in \! \mathscr {D}\big (H_{N}^{(\textrm{F})}\big )$. For any $\psi _{1},\psi _{2}\! \in \! \mathscr {D}\big [Q_{N}^{(\textrm{F})}\big ]$, integrating by parts and noting that $\textbf{A}_{n} \cdot (\textbf{x} - \textbf{x}_{n}) = 0$, we obtain

$$\begin{aligned} Q_{N}^{(\textrm{F})}[\psi _{1},\psi _{2}]&= \lim _{r \rightarrow 0^+}\! \int _{\mathbb {R}^2 \,\setminus \, \cup _{n = 1}^N B_{r}(\textbf{x}_n)}\hspace{-0.5cm}\textrm{d}\textbf{x}\; \left[ \left( -i \nabla + \textstyle \sum _{\ell \,=\, 1}^N \textbf{A}_{\ell }\right) \psi _{1} \right]^* \\&\quad \cdot \left( -i \nabla + \textstyle \sum _{m \,=\, 1}^N \textbf{A}_{m}\right) \psi _{2} \\&= \lim _{r \rightarrow 0^+}\! \left[ \int _{\mathbb {R}^2 \,\setminus \, \cup _{n = 1}^N B_{r}(\textbf{x}_n)}\hspace{-0.9cm}\textrm{d}\textbf{x}\; \psi _{1}^{*} \left( -i \nabla \!+\! \textstyle \sum _{m \,=\, 1}^N \textbf{A}_{m}\right) ^2\! \psi _{2}\right. \\&\quad \left. - i \sum _{n \,=\, 1}^N \int _{\partial B_{r}(\textbf{x}_n)}\hspace{-0.6cm}\textrm{d}\Sigma _r(\textbf{x})\; \psi _{1}^{*}\; \tfrac{\textbf{x} - \textbf{x}_{n}}{|\textbf{x} - \textbf{x}_{n}|} \cdot \left( -i\nabla + \textbf{S}_{n}\right) \psi _{2} \right] . \end{aligned}$$

Concerning the boundary terms, using the Cauchy–Schwarz inequality and the relations in (1.11), we inferSumming up, the above results show that $H_{N}^{(\textrm{F})} \psi _{1} = H_{N} \psi _{1}$, which in turn yields the statement. $\square$

We now address the proof of the representation (1.30) of the Friedrichs operator resolvent, which is going to play a very important role in the sequel. We henceforth denote by $\left\Vert \, \cdot \, \right\Vert_{\mathcal {L}^n}: = \left\Vert \, \cdot \, \right\Vert_{\mathcal {L}^n(L^2(\mathbb {R}^2))}$, $n \in [1, + \infty ]$, the Schatten norm of order *n*, and by $\mathcal {L}^n: = \mathcal {L}^n(L^2(\mathbb {R}^2))$ the corresponding operators ideal.

#### Proposition 2.1

(Resolvent of $H^{(\textrm{F})}_{N}$). For any $z \in \mathbb {C} \setminus [0,+\infty )$, the operator $T_{N}(z)$ defined in (1.32) is bounded and (1.30) holds true, i.e.,

$$\begin{aligned} R^{(\textrm{F})}_{N}(z) = \sum _{n = 0}^{N} e^{- i \textbf{S}_{n}(\textbf{x}_n) \cdot (\textbf{x} - \textbf{x}_n)}\xi _{n}\, R^{(\textrm{F})}_{n}(z)\, \xi _{n}\,e^{i \textbf{S}_{n}(\textbf{x}_n) \cdot (\textbf{x} - \textbf{x}_n)}\, \big [1 + T_N(z)\big ]^{-1} . \end{aligned}$$

We first present an auxiliary lemma. Recall the definition (1.32) of the operator $T_N(z)$:

$$\begin{aligned} T_N(z) = \textstyle \sum _{n} e^{-i \textbf{S}_{n}(\textbf{x}_n) \cdot (\textbf{x} - \textbf{x}_n)} P_n\, R^{(\textrm{F})}_{n}(z)\, \xi _{n}\,e^{i \textbf{S}_{n}(\textbf{x}_n) \cdot (\textbf{x} - \textbf{x}_n)}, \end{aligned}$$

where $$ P_n = 2 \,\left(\check{\textbf{S}}_{n} \xi _{n} - i \nabla \xi _{n}\right) \!\cdot \! (-i\nabla \!+\! \textbf{A}_{n}) + \check{\textbf{S}}_{n}^2 \xi _{n} + 2 \,\check{\textbf{S}}_{n}\! \cdot ( - i \nabla \xi _{n})\! - \Delta \xi _{n} $$.

#### Lemma 2.2

The operator $$ T_N(z)$$ is bounded for any $$ z \in \mathbb {C} {\setminus } \mathbb {R}^+ $$ and $$ \left\Vert T_N(z) \right\Vert < 1$$ for any $$ z \in \mathbb {C} $$ such that $$\textrm{dist}(z, \mathbb {R}^{+}) $$ is large enough.

#### Proof

Since $\sigma \big (H_{n}^{(\textrm{F})}\big ) = [0,+\infty )$, we have the bound

$$\begin{aligned} \left\Vert R^{(\textrm{F})}_{n}(z) \right\Vert \leqslant \frac{1}{ \textrm{dist}(z,\mathbb {R}^{+})}, \end{aligned}$$

which together with the fact that $\hbox {ran} \big (R^{(\textrm{F})}_{n}(z)\big ) \subset \mathscr {D}\big [Q_{N}^{(\textrm{F})}\big ]$ and the asymptotic conditions (1.11), yieldsSince $\xi _{n}$ is smooth and uniformly bounded together with all its derivatives, and the same can be said for $\check{\textbf{S}}_n$ on $\textrm{supp}\,\xi _{n}$, from the above arguments we readily infer^1^

$$\begin{aligned} \left\Vert T_n(z) \right\Vert \leqslant \frac{C}{\textrm{dist}(z,\mathbb {R}^{+})} \left( 1 + \frac{|z|}{\textrm{dist}(z,\mathbb {R}^{+})} \right) , \end{aligned}$$

for all $z \in \mathbb {C} \setminus [0,+\infty )$, which proves the statement. $\square$

#### Proof of Proposition 2.1

We start from the ansatz

2.1 $$\begin{aligned} R^{(\textrm{F})}_{N}(z) = \sum _{n \,=\, 0}^{N} \, e^{- i \textbf{S}_{n}(\textbf{x}_n) \cdot (\textbf{x} - \textbf{x}_n)}\xi _{n}\, R^{(\textrm{F})}_{n}(z)\, \xi _{n}\, e^{i \textbf{S}_{n}(\textbf{x}_n) \cdot (\textbf{x} - \textbf{x}_n)} + W_N(z),\nonumber \\ \end{aligned}$$

where $W_{N}(z)$ is a suitable reminder operator to be determined *a posteriori*. Applying $H_{N}^{(\textrm{F})} - z$ on both sides of (2.1), we get

$$\begin{aligned} 1&= \textstyle \sum \limits _{n} \, e^{- i \textbf{S}_{n}(\textbf{x}_n) \cdot (\textbf{x} - \textbf{x}_n)} \Big [ \xi _{n} \Big (\! \big (\! - i \nabla \!+\! \textbf{A}_{n} \!+\! \check{\textbf{S}}_{n} \big )^2\! - z \Big ) R^{(\textrm{F})}_{n}(z)\, \xi _{n} \\&\quad + 2 ( - i \nabla \xi _{n}) \!\cdot \! \big (\!-i\nabla \!+\! \textbf{A}_{n} \!+\! \check{\textbf{S}}_{n} \big ) R^{(\textrm{F})}_{n}(z)\, \xi _{n} - (\Delta \xi _{n})\, R^{(\textrm{F})}_{n}(z)\, \xi _{n} \Big ]\, e^{i \textbf{S}_{n}(\textbf{x}_n) \cdot (\textbf{x} - \textbf{x}_n)} \\&\quad + \big ( H_{N}^{(\textrm{F})} - z \big ) W_N(z) \\&= \textstyle \sum \limits _{n} \, e^{- i \textbf{S}_{n}(\textbf{x}_n) \cdot (\textbf{x} - \textbf{x}_n)} \Big [ \xi _{n} \big ( ( - i \nabla \!+\! \textbf{A}_{n})^2 - z\big ) + 2 \,(\check{\textbf{S}}_{n} \xi _{n} - i \nabla \xi _{n}) \!\cdot \! (-i\nabla \!+\! \textbf{A}_{n}) \\&\quad + \check{\textbf{S}}_{n}^2 \xi _{n} + 2 \,\check{\textbf{S}}_{n}\! \cdot ( - i \nabla \xi _{n})\! - \Delta \xi _{n} \Big ] R^{(\textrm{F})}_{n}(z)\, \xi _{n}\,e^{i \textbf{S}_{n}(\textbf{x}_n) \cdot (\textbf{x} - \textbf{x}_n)}\\&\quad + \big ( H_{N}^{(\textrm{F})}\! - z \big ) W_N(z)\,. \end{aligned}$$

Since $(( - i \nabla \!+\! \textbf{A}_{n})^2 - z ) R^{(\textrm{F})}_{n}(z) = 1$ and $\sum \nolimits _{n = 0}^{N} \xi _{n}^2 = 1$, we infer that

$$\begin{aligned} W_N(z)&= -\,R^{(\textrm{F})}_{N}(z) \; \textstyle \sum \limits _{n} \, e^{-i \textbf{S}_{n}(\textbf{x}_n) \cdot (\textbf{x} - \textbf{x}_n)} \Big [2 \,(\check{\textbf{S}}_{n} \xi _{n} - i \nabla \xi _{n}) \!\cdot \! (-i\nabla \!+\! \textbf{A}_{n}) \\&\quad + \check{\textbf{S}}_{n}^2 \xi _{n} + 2 \,\check{\textbf{S}}_{n}\! \cdot ( - i \nabla \xi _{n})\! - \Delta \xi _{n} \Big ] R^{(\textrm{F})}_{n}(z)\, \xi _{n}\, e^{i \textbf{S}_{n}(\textbf{x}_n) \cdot (\textbf{x} - \textbf{x}_n)}\,. \end{aligned}$$

Summing up, we obtain

$$\begin{aligned} R^{(\textrm{F})}_{N}(z) \big [ 1 + T_N(z) \big ] = \textstyle \sum \limits _{n} \, e^{-i \textbf{S}_{n}(\textbf{x}_n) \cdot (\textbf{x} - \textbf{x}_n)} \xi _{n}\, R^{(\textrm{F})}_{n}(z)\, \xi _{n}\,e^{i \textbf{S}_{n}(\textbf{x}_n) \cdot (\textbf{x} - \textbf{x}_n)} , \end{aligned}$$

and it just remains to point out that, due to Lemma 2.2, $1 + T_N(z)$ is invertible via the Neumann series

$$\begin{aligned} \big [1 + T_N(z)\big ]^{-1} = \sum _{j = 0}^{+\infty }\, (-1)^{j} \big (T_N(z)\big )^{j} . \end{aligned}$$

$$\square $$

We now address the spectral and scattering properties of the Friedrichs realization, which will serve as basic ingredients of the proofs of Proposition 1.8 and Proposition 1.9.

#### Proposition 2.3

(Spectral properties of $H_N^{(\textrm{F})}$). Let $N \in \mathbb {N}$ and, for all $n = 1,\dots ,N$, let $\alpha _n \in (0,1)$. Then,

$$\begin{aligned} \sigma _{\textrm{ess}}\big (H_{N}^{(\textrm{F})}\big ) = [0,+\infty ), \qquad \sigma _{\textrm{disc}}\big (H_{N}^{(\textrm{F})}\big ) = \varnothing . \end{aligned}$$

#### Proposition 2.4

(Scattering properties of $H_N^{(\textrm{F})}$). Let $N \in \mathbb {N}$ and, for all $n = 1, \ldots , N$, let $\alpha _n \in (0,1)$. Then, the wave operators $\Omega _{\pm }\big (H_{N}^{(\textrm{F})}, - \Delta \big )$ exist and are complete and, in addition,

$$\begin{aligned} \sigma _{\textrm{ac}}\big (H_{N}^{(\textrm{F})}\big ) = \sigma _{\textrm{ac}}(-\Delta ) = [0,+\infty ). \end{aligned}$$

We state first some technical lemmas which will be used in the proofs of the main results. Our arguments involve an auxiliary magnetic Schrödinger operator, namely,

2.2 $$\begin{aligned} H_{\textbf{A}} = (-i \nabla + \textbf{A})^2, \end{aligned}$$

where $\textbf{A} \in C^\infty (\mathbb {R}^2,\mathbb {R}^2)$ is any given vector potential fulfilling

2.3 $$\begin{aligned} \textbf{A} = \sum _{n\,=\,1}^{N} \textbf{A}_{n} \qquad \hbox {in }\; \mathbb {R}^2 \!\setminus \! \mathcal {Q}, \end{aligned}$$

for some bounded open subset $\mathcal {Q} \subset \mathbb {R}^2$ such that $\textbf{x}_{n} \in \mathcal {Q}$ for all $n \in \{ 1,\dots ,N\}$. In particular, under these hypotheses, we certainly have

$$\begin{aligned} \textbf{A} \in L^2_{\textrm{loc}}(\mathbb {R}^2) \cap L^{\infty }(\mathbb {R}^2), \end{aligned}$$

and

$$\begin{aligned} \textbf{A}(\textbf{x}) = \left( \,\sum _{n\,=\,1}^{N} \alpha _n \right) {\textbf{x}^{\perp } \over |\textbf{x}|^2} + \mathcal {O}\!\left({1 \over |\textbf{x}|^2} \right), \qquad \hbox {for }\; |\textbf{x}| \rightarrow +\infty . \end{aligned}$$

Without loss of generality, we further assume $\textbf{A}$ to fulfill the Coulomb gauge

2.4 $$\begin{aligned} \nabla \cdot \textbf{A} = 0 \quad \text{ in } \mathbb {R}^2. \end{aligned}$$

#### Lemma 2.5

For any $\textbf{A} \in C^\infty (\mathbb {R}^2,\mathbb {R}^2)$ fulfilling (2.3) and (2.4), the operator $H_{\textbf{A}}$ defined in (2.2) is essentially self-adjoint on $C^{\infty }_{\textrm{c}}(\mathbb {R}^2)$. Its unique self-adjoint realization is the positive operator

2.5 $$\begin{aligned} H_{\textbf{A}}^{(\textrm{F})}:= (-i \nabla + \textbf{A})^2, \qquad \mathscr {D}\big (H_{\textbf{A}}^{(\textrm{F})}\big ) = H^2(\mathbb {R}^2). \end{aligned}$$

For any $z \in \mathbb {C}$ such that $\textrm{dist}(z, \mathbb {R}^{+})$ is large enough, the resolvent operator $R_{\textbf{A}}^{(\textrm{F})}(z):= \big (H_{\textbf{A}}^{(\textrm{F})} - z \big )^{-1}$ satisfies

2.6 $$\begin{aligned} R_{\textbf{A}}^{(\textrm{F})}(z)= & {} R_{0}(z) \left[1 + \left(H_{\textbf{A}}^{(\textrm{F})} + \Delta \right) R_{0}(z) \right]^{-1}\nonumber \\= & {} \left[1 + R_{0}(z) \left(H_{\textbf{A}}^{(\textrm{F})} + \Delta \right) \right]^{-1}\! R_{0}(z) . \end{aligned}$$

#### Proof

The first part of the statement follows noting that $H_{\textbf{A}}$ is a small perturbation of the free Laplacian in the sense of Kato. In fact, for any $\psi \in H^2(\mathbb {R}^2)$ and any $\varepsilon \in (0,1)$, we have

$$\begin{aligned} \left\Vert \left( H_{\textbf{A}} + \Delta \right) \psi \right\Vert_{2}{} & {} \leqslant 2\, \left\Vert \textbf{A} \right\Vert_{\infty } \left\Vert \nabla \psi \right\Vert_{2} + \left\Vert \textbf{A} \right\Vert_{\infty }^{2}\, \left\Vert\psi \right\Vert_{2}\\{} & {} \leqslant \varepsilon \, \big \Vert (-\Delta ) \psi \big \Vert _{2} + \left(\tfrac{1}{\varepsilon } + 1 \right) \left\Vert \textbf{A} \right\Vert_{\infty }^2\, \left\Vert \psi \right\Vert_{2}. \end{aligned}$$

Furthermore, $H_{\textbf{A}}^{(\textrm{F})}$ is positive definite, so that $\sigma \big (H_{\textbf{A}}^{(\textrm{F})}\big ) \subseteq [0,+\infty )$ and $R_{\textbf{A}}^{(\textrm{F})}(z)$ is a bounded operator for any $z \in \mathbb {C} \setminus [0,+\infty )$. By means of the second resolvent identity, we infer

2.7 $$\begin{aligned} R_{\textbf{A}}^{(\textrm{F})}(z) \left[ 1 + \left(H_{\textbf{A}}^{(\textrm{F})}+ \Delta \right) R_{0} (z)\right] = R_{0}(z) . \end{aligned}$$

Recalling that $\textbf{A} \in L^{\infty }(\mathbb {R}^2)$, by elementary arguments we get

$$\begin{aligned}&\left\| \left( H_{{\textbf {A}}}^{(\text {F})} + \Delta \right) R_{0}(z) \right\| = \left\| \left( 2 {\textbf {A}} \cdot (-i \nabla ) + \left| {\textbf {A}} \right| ^2\right) R_{0}(z) \right\| \\ {}&\quad \leqslant 2\, \left\| {\textbf {A}}\right\| _{\infty } \,\mathrm {ess\, sup}_{{\textbf {k}} \in \mathbb {R}^2} \left| \tfrac{{\textbf {k}}}{|{\textbf {k}}|^2 - z} \right| + \Vert {\textbf {A}}\Vert _{\infty }^2\, \mathrm {ess\, sup}_{{\textbf {k}} \in \mathbb {R}^2} \left| \tfrac{1}{|{\textbf {k}}|^2 - z} \right| \\ {}&\quad \leqslant C \left\| {\textbf {A}} \right\| _{\infty } \left( \tfrac{1}{\sqrt{|z| - \Re z}} + \tfrac{ \left\| {\textbf {A}} \right\| _{\infty }}{\text {dist}\left( z, \mathbb {R}^{+}\right) } \right) . \end{aligned}$$

From here we deduce $\big \Vert \big (H_{\textbf{A}}^{(\textrm{F})} + \Delta \big ) R_{0}(z) \big \Vert < 1$, for any *z* far enough from $\mathbb {R}^+$, which in turn ensures that $1 + \big (H_{\textbf{A}}^{(\textrm{F})} + \Delta \big )R_{0}^{(\textrm{F})}(z)$ is indeed invertible. In view of this, the first identity in (2.6) follows readily from (2.7). The second identity in (2.6) can be derived by evaluating the adjoint of the first identity, with $z^*$ in place of *z*. $\square$

#### Lemma 2.6

For any $\textbf{A} \in C^\infty (\mathbb {R}^2,\mathbb {R}^2)$ fulfilling (2.3) and (2.4), there holds

$$\begin{aligned} R_{\textbf{A}}^{(\textrm{F})}(z) - R_{0}(z) \in \mathcal {L}^{\infty }, \qquad \forall \, z \in \mathbb {C} \setminus [0,+\infty ) , \end{aligned}$$

so that

$$\begin{aligned} \sigma _{\text {ess}}\big (H_{{\textbf {A}}}^{(\text {F})}\big ) = \sigma _{\text {ess}}( - \Delta ) = [0,+\infty ), \qquad \sigma _{\text {disc}}\big (H_{{\textbf {A}}}^{(\text {F})}\big ) = \varnothing . \end{aligned}$$

#### Proof

It suffices to prove the statement for some *z* in the resolvent set. Let us fix $z = - \lambda ^2$, for some $\lambda > 0$, and proceed to notice that the second resolvent identity yields

2.8 $$\begin{aligned} R_{\textbf{A}}^{(\textrm{F})}(-\lambda ^2) - R_{0}(-\lambda ^2)= & {} - 2\,R_{0}(-\lambda ^2)\, \textbf{A} \cdot (-i \nabla ) R_{\textbf{A}}^{(\textrm{F})}(-\lambda ^2) \nonumber \\{} & {} - R_{0}(-\lambda ^2) \left| \textbf{A} \right|^2 R_{\textbf{A}}^{(\textrm{F})}(-\lambda ^2). \end{aligned}$$

Hereafter, we show that both addenda on the right-hand side of (2.8) are indeed compact operators.

We introduce the one-parameter family of smeared vector potentials

$$\begin{aligned} \textbf{A}_{\varepsilon }(\textbf{x}):= {1 \over (1 + |\textbf{x}|)^{\varepsilon }}\,\textbf{A}(\textbf{x}) \qquad (\varepsilon > 0). \end{aligned}$$

Since $|\textbf{A}(\textbf{x})| \leqslant {C \over 1+|\textbf{x}|}$, we haveproving that $R_{0}(-\lambda ^2)\, \textbf{A}_{\varepsilon }$ is a Hilbert–Schmidt operator. To say more, we have

$$\begin{aligned} \left\Vert\textbf{A}_{\varepsilon } - \textbf{A}\right\Vert_{\infty } \leqslant C \,\mathrm{ess\, sup}_{\textbf{x} \in \mathbb {R}^2} \tfrac{(1 + |\textbf{x}|)^{\varepsilon } - 1}{(1 + |\textbf{x}|)^{\varepsilon } (1 + |\textbf{x}|)} = C \,\varepsilon \,(1+ \varepsilon )^{-{1 + \varepsilon \over \varepsilon }} \,\xrightarrow [\varepsilon \rightarrow 0^{+}]{}\, 0, \end{aligned}$$

which implies, in turn, that $R_{0}(-\lambda ^2)\, \textbf{A}_{\varepsilon }$ converges to $R_{0}(-\lambda ^2)\, \textbf{A}$ in norm. Since $(-i \nabla ) R_{\textbf{A}}^{(\textrm{F})}(-\lambda ^2)$ is bounded, we deduce that the first operator on the r.h.s. of (2.8) is compact. On the other hand,Hence, since $R_{\textbf{A}}^{(\textrm{F})}(-\lambda ^2)$ is bounded, the second operator on the r.h.s. of (2.8) is compact too.

A straightforward application of Weyl’s criterion [69, Thm. XIII.14] completes the proof. $\square$

Next, we recall an important result about scattering theory for magnetic Schrödinger operators originally proved in [49] and [74].

#### Proposition 2.7

For any $\textbf{A} \in C^\infty (\mathbb {R}^2,\mathbb {R}^2)$ fulfilling (2.3) and (2.4), the wave operators $\Omega _{\pm } (H_{\textbf{A}}^{(\textrm{F})}, - \Delta )$ exist and are asymptotically complete, so that

$$\begin{aligned} \sigma _{\textrm{ac}}\big (H_{\textbf{A}}^{(\textrm{F})}\big ) = \sigma _{\textrm{ac}}\left(-\Delta \right) = [0,+\infty ), \qquad \sigma _{\textrm{sc}}\big ( H_{\textbf{A}}^{(\textrm{F})}\big ) = \varnothing . \end{aligned}$$

#### Proof

See, e.g., [49, Theorem 2]. $$\square $$

#### Lemma 2.8

Let $N \in \mathbb {N}$ and, for all $n = 1, \ldots , N$, let $\alpha _n \in (0,1)$. Then, for any $\textbf{A} \in C^\infty (\mathbb {R}^2,\mathbb {R}^2)$ fulfilling (2.3) and (2.4), there holds

$$\begin{aligned} R_{N}^{(\textrm{F})}(z) - R_{\textbf{A}}^{(\textrm{F})}(z) \in \mathcal {L}^{2}, \qquad \forall \, z \in \mathbb {C} \setminus [0,+\infty ) . \end{aligned}$$

#### Proof

Let $\mathbb {1}_{\mathcal {Q}}$ be the indicator function associated to the bounded set $\mathcal {Q}$ appearing in (2.3). An elementary computation shows that

2.9 $$\begin{aligned} H_{N}^{(\textrm{F})} - H_{\textbf{A}}^{(\textrm{F})} = \left( {\sum _n}\textbf{A}_n - \textbf{A} \right) \cdot \big [ 2 (- i\nabla ) + {\sum _n}\textbf{A}_n + \textbf{A} \big ] = \mathbb {1}_{\mathcal {Q}} \left(H_{N}^{(\textrm{F})} - H_{\textbf{A}}^{(\textrm{F})} \right).\nonumber \\ \end{aligned}$$

Then, using the second resolvent identity and the representation (2.6) for $R_{\textbf{A}}^{(\textrm{F})}(z)$, we deduce

$$\begin{aligned}{} & {} R_{N}^{(\textrm{F})}(z) - R_{\textbf{A}}^{(\textrm{F})}(z)\\{} & {} \quad = - \left[1 + R_{0} (z) \left(H_{\textbf{A}}^{(\textrm{F})} + \Delta \right) \right]^{-1} R_{0} (z)\, \mathbb {1}_{\mathcal {Q}}\, \left( H_{N}^{(\textrm{F})} - H_{\textbf{A}}^{(\textrm{F})} \right) R_{N}^{(\textrm{F})}(z). \end{aligned}$$

Notice that $\left[\mathbb {1}+ R_{0} (z) \left(H_{\textbf{A}}^{(\textrm{F})} + \Delta \right) \right]^{-1}$ and $\big [ H_{N}^{(\textrm{F})} - H_{\textbf{A}}^{(\textrm{F})} \big ] R_{N}^{(\textrm{F})}(z)$ are bounded operators. On the other hand,

$$\begin{aligned} \left\Vert R_{0}(-\lambda ^2) \mathbb {1}_{\mathcal {Q}}\right\Vert_{\mathcal {L}^2}^{2} \leqslant \left\Vert R_{0}(-\lambda ^2) \right\Vert_2^2 \left| \Omega \right| < + \infty . \end{aligned}$$

This suffices to infer that $R_{0}^{(\textrm{F})}(z)\, \mathbb {1}_{\mathcal {Q}} \in \mathcal {L}^2$ for $z = -\lambda ^2$, whence for any $z \in \mathbb {C} \setminus [0,+\infty )$, which in turn implies the statement. $\square$

#### Lemma 2.9

Let $N \in \mathbb {N}$ and, for all $n = 1, \ldots , N$, let $\alpha _n \in (0,1)$. Then, for any $\textbf{A} \in C^\infty (\mathbb {R}^2,\mathbb {R}^2)$ fulfilling (2.3) and (2.4), there holds

$$\begin{aligned} \big (R_{N}^{(\textrm{F})}(z)\big )^2 - \big (R_{\textbf{A}}^{(\textrm{F})}(z)\big )^2 \in \mathcal {L}^1, \qquad \forall \, z \in \mathbb {C} \setminus [0,+\infty ). \end{aligned}$$

#### Proof

To begin with, let us draw the attention to the elementary algebraic identity

$$\begin{aligned}&\big (R_{N}^{(\textrm{F})}(z)\big )^2 - \big (R_{\textbf{A}}^{(\textrm{F})}(z)\big )^2\\&\quad = \left(R_{N}^{(\textrm{F})}(z) - R_{\textbf{A}}^{(\textrm{F})}(z)\right)^2\\&\qquad + \left(R_{N}^{(\textrm{F})}(z) - R_{\textbf{A}}^{(\textrm{F})}(z)\right) R_{\textbf{A}}^{(\textrm{F})}(z)\\&\qquad + R_{\textbf{A}}^{(\textrm{F})}(z) \left(R_{N}^{(\textrm{F})}(z) - R_{\textbf{A}}^{(\textrm{F})}(z)\right) . \end{aligned}$$

Since $R_{N}^{(\textrm{F})}(z) - R_{\textbf{A}}^{(\textrm{F})}(z) \in \mathcal {L}^2$ by Lemma 2.8, using basic properties of Hilbert–Schmidt operators we readily obtain that $\big (R_{N}^{(\textrm{F})}(z) - R_{\textbf{A}}^{(\textrm{F})}(z)\big )^2 \in \mathcal {L}^1$ (see, e.g., [69, Thm. VI.22]). For *z* real the other two terms are one the adjoint of the other, so it suffices to prove that

$$\begin{aligned} R_{\textbf{A}}^{(\textrm{F})}(z) \left(R_{N}^{(\textrm{F})}(z) - R_{\textbf{A}}^{(\textrm{F})}(z)\right) \in \mathcal {L}^1. \end{aligned}$$

Let $\chi _{\mathcal {Q}} \in C^{\infty }_{\textrm{c}}(\mathbb {R}^2)$ be such that $\chi _{\mathcal {Q}} \equiv 1$ in $\mathcal {Q}$. Acting as in the derivation of (2.9), we get

$$\begin{aligned}{} & {} R_{\textbf{A}}^{(\textrm{F})}(z) \left(R_{N}^{(\textrm{F})}(z) - R_{\textbf{A}}^{(\textrm{F})}(z)\right)\\{} & {} \qquad = -\, \big (R_{\textbf{A}}^{(\textrm{F})}(z)\big )^2\, \chi _{\mathcal {Q}} \left(H_{N}^{(\textrm{F})} - H_{\textbf{A}}^{(\textrm{F})}\right) R_{N}^{(\textrm{F})}(z). \end{aligned}$$

Since $\big (H_{N}^{(\textrm{F})} - H_{\textbf{A}}^{(\textrm{F})}\big ) R_{N}^{(\textrm{F})}(z)$ is a bounded operator, it remains to show that

2.10 $$\begin{aligned} \big (R_{\textbf{A}}^{(\textrm{F})}(z)\big )^2\, \chi _{\mathcal {Q}} \in \mathcal {L}^1 . \end{aligned}$$

To this purpose, we repeatedly exploit the following identity, which is a straightforward consequence of the Coulomb gauge,

$$\begin{aligned} \left[(-i\partial _{j}), H_{\textbf{A}}^{(\textrm{F})} \right]&= \left[(-i\partial _{j}), \left|\textbf{A}\right|^2 \right] + 2 \sum _{k = 1}^2 \left[(-i\partial _{j}), \textbf{A}_k \right] (-i\partial _k)\\&= - i \partial _j \left| \textbf{A} \right|^2 + 2 \sum _{k = 1}^2 (-i \partial _j \textbf{A}_k) (-i\partial _k) \\&= - i \partial _j \left| \textbf{A} \right|^2 + 2 \left(- i \nabla \right) \cdot (-i \partial _j \textbf{A})\,, \end{aligned}$$

to compute

2.11 $$\begin{aligned}&\big (R_{\textbf{A}}^{(\textrm{F})}(z)\big )^2 \chi _{\mathcal {Q}} = - \,\big (R_{\textbf{A}}^{(\textrm{F})}(z)\big )^2\big [H_{\textbf{A}}^{(\textrm{F})}, \chi _{\mathcal {Q}}\big ] R_{\textbf{A}}^{(\textrm{F})}(z) + R_{\textbf{A}}^{(\textrm{F})}(z)\,\chi _{\mathcal {Q}} \, R_{\textbf{A}}^{(\textrm{F})}(z) \nonumber \\&\quad = - \,2 \big (R_{\textbf{A}}^{(\textrm{F})}(z)\big )^2 (-i \nabla ) \cdot (- i \nabla \chi _{\mathcal {Q}}) R_{\textbf{A}}^{(\textrm{F})}(z) \nonumber \\&\qquad -\, \big (R_{\textbf{A}}^{(\textrm{F})}(z)\big )^2 \big [2 \textbf{A} \cdot (- i \nabla \chi _{\mathcal {Q}}) + \Delta \chi _{\mathcal {Q}} \big ] R_{\textbf{A}}^{(\textrm{F})}(z)+ R_{\textbf{A}}^{(\textrm{F})}(z)\,\chi _{\mathcal {Q}} \, R_{\textbf{A}}^{(\textrm{F})}(z) \nonumber \\&\quad = - \,4 \sum _{j =1}^2 \left\{ \big (R_{\textbf{A}}^{(\textrm{F})}(z) \big )^2 \big [ 2 (-i \partial _j \textbf{A}) \cdot (-i \nabla )\right. \nonumber \\&\qquad + \big (- i \partial _j \left|\textbf{A}\right|^2\big ) \big ] R_{\textbf{A}}^{(\textrm{F})}(z) (-i \nabla \textbf{A}_j) \cdot R_{\textbf{A}}^{(\textrm{F})}(z) (- i \nabla \chi _{\mathcal {Q}}) R_{\textbf{A}}^{(\textrm{F})}(z) \nonumber \\&\qquad + \,2\, R_{\textbf{A}}^{(\textrm{F})}(z) \big [ 2 (-i\partial _j) (-i \nabla \textbf{A}_j) + \big (- i \nabla \left|\textbf{A}\right|^2\big )\big ] \cdot \big (R_{\textbf{A}}^{(\textrm{F})}(z) \big )^2 (- i \nabla \chi _{\mathcal {Q}}) R_{\textbf{A}}^{(\textrm{F})}(z) \nonumber \\&\qquad \left. -\,4\, R_{\textbf{A}}^{(\textrm{F})}(z) (-i\partial _j) R_{\textbf{A}}^{(\textrm{F})}(z)\, (-i \nabla \textbf{A}_j) \cdot R_{\textbf{A}}^{(\textrm{F})}(z) (- i \nabla \chi _{\mathcal {Q}}) R_{\textbf{A}}^{(\textrm{F})}(z) \right\} \nonumber \\&\qquad -\,2 \big (R_{\textbf{A}}^{(\textrm{F})}(z)\big )^2 \big (- i \nabla \left|\textbf{A}\right|^2 \big ) \cdot \, R_{\textbf{A}}^{(\textrm{F})}(z) (- i \nabla \chi _{\mathcal {Q}}) R_{\textbf{A}}^{(\textrm{F})}(z)\nonumber \\&\qquad - \,2\, (-i \nabla ) \cdot \big (R_{\textbf{A}}^{(\textrm{F})}(z)\big )^2 (- i \nabla \chi _{\mathcal {Q}}) R_{\textbf{A}}^{(\textrm{F})}(z)\nonumber \\&\qquad -\, \big (R_{\textbf{A}}^{(\textrm{F})}(z)\big )^2 \big [2 \textbf{A} \cdot (- i \nabla \chi _{\mathcal {Q}}) + \Delta \chi _{\mathcal {Q}} \big ] R_{\textbf{A}}^{(\textrm{F})}(z) + R_{\textbf{A}}^{(\textrm{F})}(z)\,\chi _{\mathcal {Q}} \, R_{\textbf{A}}^{(\textrm{F})}(z)\,. \end{aligned}$$

Given that $\textbf{A}$, $\chi$ and all their derivatives are uniformly bounded in $\mathbb {R}^2$, they all define bounded operators. Furthermore, since $\mathscr {D}\big (H_{\textbf{A}}^{(\textrm{F})}\big ) = H^2(\mathbb {R}^2)$ (see (2.5)), we certainly have $(-i \nabla ) R_{\textbf{A}}^{(\textrm{F})}(z) \in \mathcal {B}(L^2(\mathbb {R}^2))$. Taking this into account, it can be checked by direct inspection that each addendum in the last expression of (2.11) is indeed a product of bounded operators including at least one term of the form

$$\begin{aligned} R_{\textbf{A}}^{(\textrm{F})}(z)\,\mathcal {X}\, R_{\textbf{A}}^{(\textrm{F})}(z) , \qquad \text{ for } \text{ some } \mathcal {X} \in C^{\infty }_{\textrm{c}}(\mathbb {R}^2). \end{aligned}$$

We claim that any such term belongs to $\mathcal {L}^1$. As a matter of fact, in view of (2.6), it suffices to prove that

2.12 $$\begin{aligned} R_{0}(-\lambda ^2)\,\mathcal {X}\, R_{0}(-\lambda ^2) = R_{0}(-\lambda ^2)\,\mathbb {1}_{\textrm{supp}\,\mathcal {X}}\,\mathcal {X}\, \mathbb {1}_{\textrm{supp}\,\mathcal {X}} R_{0}(-\lambda ^2) \in \mathcal {L}^1, \end{aligned}$$

for some $\lambda > 0$ large enough. In this connection, we notice that

$$\begin{aligned} \left\Vert R_{0}(-\lambda ^2)\,\mathbb {1}_{\textrm{supp}\,\mathcal {X}} \right\Vert_{\mathcal {L}^2}^2 \! \leqslant \left\Vert R_0 \right\Vert_2^2\, \left| \textrm{supp}\,\mathcal {X} \right| < + \infty , \end{aligned}$$

with a completely analogous bound for the adjoint operator. Hence, since $\mathcal {X}$ is bounded, we conclude that (2.12) holds by [69, Thm. VI.22], which in turn implies (2.10), whence the statement. $\square$

We are now able to prove the characterization of the spectrum of $H_N^{(\textrm{F})}$ and of its scattering w.r.t. the free Laplacian.

#### Proof of Proposition 2.3

Consider any auxiliary operator $H_{\textbf{A}}^{(\textrm{F})}$, with $\textbf{A}$ fulfilling (2.3) and (2.4). By Lemma 2.6 and Lemma 2.8, we readily infer that

$$\begin{aligned}{} & {} R_{N}^{(\textrm{F})}(z) - R_{0}(z)\\{} & {} \quad = R_{N}^{(\textrm{F})}(z) - R^{(\textrm{F})}_{\textbf{A}}(z) + R^{(\textrm{F})}_{\textbf{A}}(z) - R_{0}(z) \in \mathcal {L}^{\infty }, \quad \forall \, z \in \mathbb {C} \setminus [0,+\infty ). \end{aligned}$$

Then, recalling that $H_{N}^{(\textrm{F})}$ is nonnegative, the statement follows by Weyl’s criterion. $\square$

#### Proof of Proposition 2.4

Let again $H_{\textbf{A}}^{(\textrm{F})}$ be any auxiliary operator, with $\textbf{A}$ fulfilling (2.3) and (2.4). By Lemma 2.9 and the invariance principle [69, Corollary 3 (vol. III, p. 30)], we deduce the existence and completeness of the wave operators

$$\begin{aligned} \Omega _{\pm }\left(H_{N}^{(\textrm{F})},H_{\textbf{A}}^{(\textrm{F})}\right):= \lim \limits _{t \rightarrow \mp \infty } e^{i t H_{N}^{(\textrm{F})}} e^{-i t H_{\textbf{A}}^{(\textrm{F})}}. \end{aligned}$$

Then, on account of Proposition 2.7, the statement follows by the chain rule [69, Proposition 2 (vol. III, p. 18)]. $\square$

### Singular Perturbations: Quadratic Forms

For later reference, we first observe that the sesquilinear form defined by polarization starting from $Q_{N}^{(B)}$ w.r.t. the decompositions

$$\begin{aligned} \psi _j = \phi _{j,\lambda } + \textstyle \sum \limits _{n} \, e^{-i \textbf{S}_{n}(\textbf{x}_n) \cdot (\textbf{x} - \textbf{x}_n)}\, \xi _{n}\, \textstyle \sum \limits _{\ell _n} \, q^{(\ell _n)}_{j,n}\, G^{(\ell _n)}_{\lambda ,n} \qquad (j = 1,2 ) \end{aligned}$$

is given by

2.13 $$\begin{aligned}&Q^{(B)}_{N}[\psi _1,\psi _2]= Q^{(\textrm{F})}_{N}[\phi _{1,\lambda },\phi _{2,\lambda }] - \lambda ^2\, \langle \psi _1\,|\,\psi _2\rangle + \lambda ^2\,\langle \phi _{1,\lambda }\,|\,\phi _{2,\lambda }\rangle \nonumber \\&\quad + \textstyle \sum \limits _{n,\ell _n} \big ({q_{1,n}^{(\ell _n)}}\big )^* \bigg ( 2 \left.\left\langle \,e^{-i \textbf{S}_{n}(\textbf{x}_n) \cdot (\textbf{x} - \textbf{x}_n)} \big (\check{\textbf{S}}_{n}\,\xi _{n} - i \nabla \xi _{n} \big ) G^{(\ell _n)}_{\lambda ,n}\right| \left(-i \nabla \!+\! \textbf{A}_{n}\right)\phi _{2,\lambda } \right\rangle \nonumber \\&\quad + \left.\left\langle e^{-i \textbf{S}_{n}(\textbf{x}_n) \cdot (\textbf{x} - \textbf{x}_n)}\left( \check{\textbf{S}}_{n}^2\, \xi _{n} + 2\, \textbf{S}_n(\textbf{x}_n) \cdot (\check{\textbf{S}}_n \xi _{n} - i \nabla \xi _{n}) + \Delta \xi _{n} \right)\! G^{(\ell _n)}_{\lambda ,n}\right|\phi _{2,\lambda } \right\rangle \bigg ) \nonumber \\&\quad + \textstyle \sum \limits _{n,\ell _n} q^{(\ell _n)}_{2,n} \bigg ( 2 \left\langle \left(-i \nabla \!+\! \textbf{A}_{n}\right)\phi _{1,\lambda }\left|\,e^{-i \textbf{S}_{n}(\textbf{x}_n) \cdot (\textbf{x} - \textbf{x}_n)} \big (\check{\textbf{S}}_{n}\,\xi _{n} - i \nabla \xi _{n} \big ) G^{(\ell _n)}_{\lambda ,n}\right. \right\rangle \nonumber \\&\quad + \left\langle \phi _{1,\lambda }\left|e^{-i \textbf{S}_{n}(\textbf{x}_n) \cdot (\textbf{x} - \textbf{x}_n)}\left( \check{\textbf{S}}_{n}^2\, \xi _{n} + 2\, \textbf{S}_n(\textbf{x}_n) \cdot (\check{\textbf{S}}_n \xi _{n} - i \nabla \xi _{n}) + \Delta \xi _{n} \right)\! G^{(\ell _n)}_{\lambda ,n}\right. \right\rangle \bigg ) \nonumber \\&\quad + \textstyle \sum \limits _{m, n, \ell _m, \ell _n^{\prime }} \big ({q^{(\ell _m)}_{1,m}}\big )^* q^{(\ell _n^{\prime })}_{2,n} \left[ B^{(\ell _m \ell _n^{\prime })}_{m\,n} + \delta _{m n} \left( \tfrac{\pi \,\lambda ^{2|\ell _m + \alpha _m|}}{2 \sin (\pi \alpha _m)}\,\delta _{\ell _m \ell _n^{\prime }} + \Xi ^{(\ell _m \ell _n^{\prime })}_{n}(\lambda ) \right) \right] . \end{aligned}$$

#### Proof of Theorem 1.3

*i)* Let us first prove that the quadratic form (1.17) is independent of $\lambda > 0$. To this avail, we fix $\lambda _1 \ne \lambda _2$ and consider, for any $\psi \in \mathscr {D}\big [Q^{(B)}_{N}\big ]$, the alternative representations

$$\begin{aligned} \psi = \phi _{\lambda _j} \!+ \sum \limits _{n,\ell _n} e^{-i \textbf{S}_{n}(\textbf{x}_n) \cdot (\textbf{x} - \textbf{x}_n)}\,\xi _{n} q^{(\ell _n)}_{n} G^{(\ell _n)}_{\lambda _j,n} \qquad (j =1,2 ). \end{aligned}$$

Since $\xi _{n}(G^{(\ell _n)}_{\lambda _2,n} - G^{(\ell _n)}_{\lambda _1,n}) \in \mathscr {D}\big [Q_{N}^{(\textrm{F})}\big ]$, for all $n \in \{1,\dots ,N\}$, we deduce that

$$\begin{aligned} \phi _{\lambda _1} = \phi _{\lambda _2} \!+ {\sum \limits _{n,\ell _n}} e^{-i \textbf{S}_{n}(\textbf{x}_n) \cdot (\textbf{x} - \textbf{x}_n)}\,\xi _{n} q^{(\ell _n)}_{n} \big ( G^{(\ell _n)}_{\lambda _2,n} - G^{(\ell _n)}_{\lambda _1,n}\big ) \end{aligned}$$

and thus the “charges” $q^{(\ell _n)}_{n}$ are independent of $\lambda$. Furthermore, by using (1.13), the identity $\textbf{A}_n \cdot (\textbf{x} - \textbf{x}_n) = 0$ and the fact that $\xi _{n}$ is real-valued, we obtain, denoting for short by $\psi _j$, $j = 1,2$, the two decompositions,2.14where we dropped all the vanishing boundary terms. Since $\xi _{n} = 1$ in an open neighborhood of $\textbf{x}_n$ and thanks to (1.7), (1.11) and the asymptotic expansion (1.15), we indeed haveBy similar arguments, we deduce the following relations:On the other hand, using again the asymptotic expansion (1.15) for $G^{(\ell _n)}_{\lambda ,n}$, we getApplying the identities for the Euler gamma function in [61, Eqs. 5.5.1 and 5.5.3], we deduce thatIn view of the above arguments, the identity (2.14) reduces to

$$\begin{aligned}&Q^{(B)}_{N}\left[ \psi _1 \right] - Q^{(B)}_{N}\left[ \psi _2 \right]\\&\quad = - 2i \,\Re \! \sum \limits _{n,\ell _n,\ell _n^{\prime }}\! \big ({q^{(\ell _n)}_{n}}\big )^* q^{(\ell _n^{\prime })}_{n} \bigg (\! \left\langle G^{(\ell _n)}_{\lambda _1,n}\left|(\xi _{n}\nabla \xi _{n}) \cdot (-i \nabla \!+\! \textbf{A}_n) G^{(\ell _n^{\prime })}_{\lambda _1,n}\right. \right\rangle \\&\qquad +\, \left\langle G^{(\ell _n)}_{\lambda _2,n}\left|(\xi _{n} \nabla \xi _{n}) \cdot (-i \nabla \!+\! \textbf{A}_n) G^{(\ell _n^{\prime })}_{\lambda _2,n}\right. \right\rangle \!\bigg )\,, \end{aligned}$$

where we used that the functions $\xi _{n}$ are real-valued. Since the quadratic form $Q^{(B)}_{N}$ is real-valued as well, the latter relation can be fulfilled only if

$$\begin{aligned} Q^{(B)}_{N}\left[ \psi _1 \right] = Q^{(B)}_{N}\left[ \psi _2 \right], \end{aligned}$$

which ultimately proves that the quadratic form is independent of the spectral parameter $\lambda$.

Next, we show that the form does not depend on the cutoff functions. Let $\left(\xi _{1,n}\right)_{n \in \{1, \ldots , N\}}$, $\left(\xi _{2,n} \right)_{n \in \{1, \ldots , N\}}$ be two families of such functions. Correspondingly, for any $\psi \in \mathscr {D}\big [Q^{(B)}_{N}\big ]$, we have two alternative representations which we denote again for short $\psi _j$, $j = 1,2$. Notice that $(\xi _{2,n} - \xi _{1,n}) G^{(\ell _n)}_{\lambda ,n} \in \mathscr {D}[Q_{N}^{(\textrm{F})}]$, for all $n \in \{1,\dots ,N\}$ and $\ell _{n}\in \{0,-1\}$, so that

$$\begin{aligned} \phi _{1,\lambda } = \phi _{2,\lambda } + \sum \limits _{n,\ell _n} e^{-i \textbf{S}_{n}(\textbf{x}_n) \cdot (\textbf{x} - \textbf{x}_n)}\,\big (\chi _{2,n} - \chi _{1,n} \big ) q^{(\ell _n)}_{n} G^{(\ell _n)}_{\lambda ,n}. \end{aligned}$$

Using (1.13), by direct computations we infer

$$\begin{aligned}&Q^{(B)}_{N}\!\left[ \psi _1 \right] - Q^{(B)}_{N}\!\left[ \psi _2 \right] \\ {}&\quad = - 2i\, \Re \! \sum \limits _{n,\ell _n,\ell _n^{\prime }}\! \big ({q^{(\ell _n)}_{n}}\big )^* q^{(\ell _n^{\prime })}_{n} \left\langle {\big (\chi _{2,n} \!-\! \chi _{1,n} \big ) G^{(\ell _n)}_{\lambda ,n}}\big ( \nabla \chi _{2,n} \! - \! \nabla \chi _{1,n} \big ) \cdot (- i \nabla + {\textbf {A}}_n) G^{(\ell _n^{\prime })}_{\lambda ,n}\right\rangle , \end{aligned}$$

where all the boundary terms coming from the integration by parts vanish since $\xi _{1,n} = \xi _{2,n} = 1$ in an open neighborhood of $\textbf{x}_n$. Recalling again that the quadratic form is real, it appears that the latter relation can be fulfilled if and only if $Q^{(B)}_{N}\!\left[ \psi _1 \right] = Q^{(B)}_{N}\!\left[ \psi _2 \right]$, which proves that the form is independent of the family of cutoff functions.

*ii)* To begin with, let us show that $Q^{(B)}_{N}$ is bounded from below. In the sequel, we set $\mathbb {1}_{n}: = \mathbb {1}_{\textrm{supp}\,\xi _{n}}$ and pick $\lambda > 0$ large enough. By Cauchy inequality, for any $\epsilon \in (0,1)$ we infer

2.15 $$\begin{aligned} Q_{N}^{(\textrm{F})}[\phi _{\lambda }]&\geqslant \textstyle {1 \over 2}\, Q_{N}^{(\textrm{F})}[\phi _{\lambda }] + \textstyle {1 \over 2}\, \sum \limits _{n = 1}^{N} \left\Vert \mathbb {1}_{n} \left(-i\nabla + \textbf{A}_{n} + \textbf{S}_{n} \right)\phi _{\lambda } \right\Vert_{2}^{2} \nonumber \\&\geqslant \textstyle {1 \over 2}\, Q_{N}^{(\textrm{F})}[\phi _{\lambda }] + \textstyle \frac{1 - \epsilon }{2}\, \sum \limits _{n = 1}^{N} \left\Vert \mathbb {1}_{n} \left(-i\nabla + \textbf{A}_{n} \right)\phi _{\lambda } \right\Vert_{2}^{2}\nonumber \\&\quad - \textstyle \frac{1 - \epsilon }{2\epsilon }\, \sum \limits _{n = 1}^{N} \left\Vert \mathbb {1}_{n} \textbf{S}_{n} \right\Vert_{\infty }^2\,\Vert \phi _{\lambda }\Vert _{2}^{2}\,. \end{aligned}$$

Furthermore, for any $\epsilon _1, \epsilon _2 \in (0,1)$ we have

2.16 $$\begin{aligned}&\Re \left[ q^{(\ell _n)}_{n} \left\langle \left(-i \nabla + \textbf{A}_{n} \right)\phi _{\lambda }\left|\,\,e^{-i \textbf{S}_{n}(\textbf{x}_n) \cdot (\textbf{x} - \textbf{x}_n)} \big (\check{\textbf{S}}_{n}\,\xi _{n} - i \nabla \xi _{n} \big ) G^{(\ell _n)}_{\lambda ,n}\right. \right\rangle \right] \nonumber \\&\quad \geqslant -\, \textstyle {\epsilon _1 \over 2}\, \big \Vert \mathbb {1}_{n} (-i \nabla + \textbf{A}_{n}) \phi _{\lambda } \big \Vert _{2}^{2} - \textstyle {1 \over 2\epsilon _1} \left|q_{n}^{(\ell _n)}\right|^2\, \left\Vert \big ( \check{\textbf{S}}_{n} \,\xi _{n} - i\nabla \xi _{n} \big ) G^{(\ell _n)}_{\lambda ,n} \right\Vert_{2}^{2} \nonumber \\&\quad \geqslant -\, \textstyle {\epsilon _1 \over 2}\, \left\Vert \mathbb {1}_{n} \left(-i \nabla + \textbf{A}_{n}\right) \phi _{\lambda } \right\Vert_{2}^{2} - \textstyle {C \over \epsilon _1} \left|q^{(\ell _n)}_{n}\right|^2\, \lambda ^{2|\ell _n + \alpha _{n}|-2}\,; \end{aligned}$$

2.17 $$\begin{aligned}&\Re \left[ q^{(\ell _n)}_{n} \left\langle \phi _{\lambda }\left|e^{-i \textbf{S}_{n}(\textbf{x}_n) \cdot (\textbf{x} - \textbf{x}_n)}\left( \check{\textbf{S}}_{n}^2\, \xi _{n} + 2 \textbf{S}_n(\textbf{x}_n) \cdot (\check{\textbf{S}}_n \xi _{n} - i \nabla \xi _{n}) + \Delta \xi _{n} \right)\! G^{(\ell _n)}_{\lambda ,n}\right. \right\rangle \right] \nonumber \\&\quad \geqslant - \,\textstyle {\epsilon _2 \over 2}\,\Vert \mathbb {1}_{n} \phi _{\lambda }\Vert _{2}^{2} - \textstyle {C \over \epsilon _2} \left|q^{(\ell _n)}_{n}\right|^2\, \lambda ^{2|\ell _n + \alpha _{n}|-2}\,. \end{aligned}$$

Finally, let us consider the term $\Xi _{\alpha ,n}(\lambda )$ defined in (1.20). Notice that (1.7) ensues $\check{\textbf{S}}_{n}\!\cdot \!\textbf{A}_{n}\,\xi _{n}^2 \in L^{\infty }(\mathbb {R}^2)$ and $\xi _{n} \check{\textbf{S}}_n\,|\textbf{x} - \textbf{x}_{n}|^{-1} \in L^{\infty }(\mathbb {R}^2)$. Taking this into account and building on the basic inequality

$$\begin{aligned} \left||\textbf{x} - \textbf{x}_{n}|\, (-i \nabla ) \big (\xi _{n} G^{(\ell _n)}_{\lambda ,n}\big )\right| \leqslant C \left| G^{(\ell _n)}_{\lambda ,n} \right|, \end{aligned}$$

we get

2.18 $$\begin{aligned} \left|\Xi ^{(\ell _n\ell _n^{\prime })}_{n}(\lambda )\right| \leqslant C \left\Vert G^{(\ell _n)}_{\lambda ,n}\right\Vert_{2} \left\Vert G^{(\ell _n^{\prime })}_{\lambda ,n} \right\Vert_{2} \leqslant C\, \lambda ^{|\ell _n+\alpha _n| + |\ell _n^{\prime } + \alpha _n| - 2}\,. \end{aligned}$$

For any $\lambda > 0$ sufficiently large, combining (2.15)–(2.18), we estimatewhere $\left\Vert B \right\Vert_{\infty }:= \max _{m,n,\ell _m \ell _n^{\prime }}\! |B^{(\ell _m \ell _n^{\prime })}_{m\,n} |$. Now, by the positivity of $Q_{N}^{(\textrm{F})}$, if we take $\epsilon , \epsilon _2 \in (0,1)$, $0< \epsilon _1 < (1 - \epsilon )/8$ and $\lambda > 0$ large enough (notice that $\max _{n,\ell _n} |\ell _n + \alpha _n| < 1$), the above relation implies that $Q^{(B)}_{N}[\psi ] \geqslant - \lambda ^2\, \Vert \psi \Vert _{2}^{2}$, i.e., the form is bounded from below. More precisely, for any $\lambda$ large enough, we deduce that there exists $c_{\lambda } > 0$ such that

$$\begin{aligned} Q^{(B)}_{N}[\psi ] + \lambda ^2\,\Vert \psi \Vert _{2}^{2} \geqslant c_{\lambda } \left[Q_{N}^{(\textrm{F})}[\phi _{\lambda }] + \Vert \phi _{\lambda }\Vert _{2}^{2} + \left| \textbf{q} \right|^2 \right], \end{aligned}$$

i.e., the quadratic form is also coercive. This allows to infer that the form $Q^{(B)}_{N}$ is closed by classical arguments (see, e.g., [78] and [21, Proof of Thm. 2.4]).

$$\square $$

Hereafter we prove the first part of Theorem 1.4, regarding the characterization of the domain and action of the self-adjoint operators $H_{N}^{(B)}$, $B \in M_{2N,\,\textrm{Herm}}(\mathbb {C})$, corresponding to the quadratic forms $Q_{N}^{(B)}$.

#### Proof of Theorem 1.4—Part I

Consider the sesquilinear form (2.13) associated to $Q_{N}^{(B)}$. Let us first assume $\textbf{q}_1 = \textbf{0}$, i.e., $\psi _{1} = \phi _{1,\lambda }$; then, upon integration by parts, we obtain

$$\begin{aligned}&Q^{(B)}_{N}[\phi _{1,\lambda },\psi _2]= \left\langle \phi _{1,\lambda }\left|H_{N}\right|\phi _{2,\lambda }\right\rangle \\&\qquad + \sum \limits _{n,\ell _n} q^{(\ell _n)}_{2,n} \bigg [2 \left\langle \phi _{1,\lambda }\left|\,e^{-i \textbf{S}_{n}(\textbf{x}_n) \cdot (\textbf{x} - \textbf{x}_n)} \big (\check{\textbf{S}}_{n}\,\xi _{n} - i \nabla \xi _{n} \big ) \left(-i \nabla \!+\! \textbf{A}_{n}\right)G^{(\ell _n)}_{\lambda ,n}\right. \right\rangle \\&\qquad + \left\langle \phi _{1,\lambda }\left|\,e^{-i \textbf{S}_{n}(\textbf{x}_n) \cdot (\textbf{x} - \textbf{x}_n)} \left( (\check{\textbf{S}}_{n}^2 - \lambda ^2)\, \xi _{n} + 2\check{\textbf{S}}_{n} \!\cdot \! (-i \nabla \xi _{n}) - \Delta \xi _{n} \right) G^{(\ell _n)}_{\lambda ,n}\right. \right\rangle \bigg ]\,, \end{aligned}$$

where again the boundary terms have been dropped, since by the asymptotic relations in Eq. (1.11) of Proposition 1.2 and arguments similar to those outlined in the proof of Theorem 1.3, we getThus, the condition $Q^{(B)}_{N}[\phi _{1,\lambda },\psi _2] = \langle \phi _{1,\lambda }\,|\,w\rangle$ for some $w =: H_{N}^{(B)}\, \psi _2 \!\in \! L^2(\mathbb {R}^2)$ can be fulfilled only if $H_{N}\, \phi _{2,\lambda } \in L^2(\mathbb {R}^2)$, *i.e.*, $\phi _{2,\lambda } \in \mathscr {D}\big (H_{N}^{(\textrm{F})}\big )$, and

2.19 $$\begin{aligned} w&= H_{N}\, \phi _{2,\lambda } + \sum \limits _{n,\ell _n} q^{(\ell _n)}_{2,n}\, e^{-i \textbf{S}_{n} (\textbf{x}_n) \cdot (\textbf{x} - \textbf{x}_n)} \left[ 2 \big (\check{\textbf{S}}_{n}\,\xi _{n} - i \nabla \xi _{n} \big ) \left(-i \nabla \!+\! \textbf{A}_{n}\right)G^{(\ell _n)}_{\lambda ,n} \right. \nonumber \\&\quad \left. + \left( (\check{\textbf{S}}_{n}^2 - \lambda ^2)\, \xi _{n} + 2\check{\textbf{S}}_{n} \!\cdot \! (-i \nabla \xi _{n}) - \Delta \xi _{n} \right) G^{(\ell _n)}_{\lambda ,n} \right] . \end{aligned}$$

Let now assume that $\textbf{q} \ne \textbf{0}$. Integrating by parts and checking again that the boundary contributions vanish, we get

$$\begin{aligned}&Q^{(B)}_{N}[\psi _1,\psi _2] = Q^{(B)}_{N}[\phi _{1,\lambda },\psi _2] \\&\quad + \sum \limits _{m,\ell _m} \big (q_{1,m}^{(\ell _m)}\big )^* \left( 2 \left.\left\langle \,e^{-i \textbf{S}_{m}(\textbf{x}_m) \cdot (\textbf{x} - \textbf{x}_m)} \big (\check{\textbf{S}}_{m}\,\xi _{m} - i \nabla \xi _{m} \big ) G^{(\ell _m)}_{\lambda ,m}\right| \left(-i \nabla \!+\! \textbf{A}_{m}\right)\phi _{2,\lambda } \right\rangle \right. \\&\quad \left. + \left.\left\langle e^{-i \textbf{S}_{m}(\textbf{x}_m) \cdot (\textbf{x} - \textbf{x}_m)}\!\left( (\check{\textbf{S}}_{m}^2\! - \lambda ^2)\, \xi _{m} + 2\, \textbf{S}_{m}(\textbf{x}_m) \cdot (\check{\textbf{S}}_m \xi _{m} - i \nabla \xi _{m}) + \Delta \xi _{m} \right)\! G^{(\ell _m)}_{\lambda ,m}\right| \phi _{2,\lambda } \right\rangle \right) \\&\quad + \sum \limits _{m, n,\ell _m,\ell _n^{\prime }} \big ({q^{(\ell _m)}_{1,m}}\big )^* q^{(\ell _n^{\prime })}_{2,n}\\&\quad \left[ B^{(\ell _m \ell _n^{\prime })}_{m\,n} + \delta _{mn} \left( \tfrac{\pi \,\lambda ^{2|\ell _n + \alpha _n|}}{2\sin (\pi \alpha _n)}\,\delta _{\ell _n \ell _n^{\prime }}\! + \Xi ^{(\ell _n \ell _n^{\prime })}_{n}(\lambda ) - \lambda ^2 \left\langle \xi _{n} G^{(\ell _n)}_{\lambda ,n}\left|\xi _{n} G^{(\ell _n^{\prime })}_{\lambda ,n}\right. \right\rangle \! \right) \right] . \end{aligned}$$

Then, demanding $Q^{(B)}_{N}[\psi _1,\psi _2] = \langle \psi _1\,|\,w\rangle$, with *w* as in (2.19), implies

2.20 $$\begin{aligned}&\sum \limits _{m, n,\ell _m,\ell _n^{\prime }} \big ({q^{(\ell _m)}_{1,m}}\big )^* q^{(\ell _n^{\prime })}_{2,n} \left[ B^{(\ell _m \ell _n^{\prime })}_{m\,n} + \delta _{mn} \left( \tfrac{\pi \,\lambda ^{2|\ell _n + \alpha _n|}}{2\sin (\pi \alpha _n)}\,\delta _{\ell _m \ell _n^{\prime }} \right.\right. \nonumber \\&\qquad - \left\langle G^{(\ell _m)}_{\lambda ,n}\left|\big [ (-i\nabla ) \!\cdot \! \big (\xi _{n} (-i\nabla \xi _{n})\big ) \big ] G^{(\ell _n^{\prime })}_{\lambda ,n} \right. \right\rangle \nonumber \\&\qquad \left. \left.- \left\langle G^{(\ell _m)}_{\lambda ,n}\left|2 \xi _{n} (-i\nabla \xi _{n}) \!\cdot \! (- i\nabla \!+\! \textbf{A}_{n}) G^{(\ell _n^{\prime })}_{\lambda ,n}\right. \right\rangle \right) \right] \nonumber \\&\quad = \sum \limits _{m,\ell _m} \big ({q^{(\ell _m)}_{1,m}}\big )^* \left\langle G^{(\ell _m)}_{\lambda ,m}\left| \left[ (-i \nabla \!+\! \textbf{A}_m)^2 + \lambda ^2 \right] e^{i \textbf{S}_{m}(\textbf{x}_m) \cdot (\textbf{x} - \textbf{x}_m)}\, \xi _{m} \phi _{2,\lambda }\right. \right\rangle . \end{aligned}$$

Integrating by parts twice and using (1.13), we obtainwhere we used that $\xi _{n} = 1$ in an open neighborhood of $\textbf{x}_{n}$ and (1.14). Concerning the r.h.s. of (2.20), by similar arguments and recalling again that $\textbf{A}_{n} \cdot (\textbf{x} - \textbf{x}_{n}) = 0$, we infer thatwhere the boundary term vanishes sinceUsing the asymptotic expansion (1.15) of $G^{(\ell _n)}_{\lambda ,n}$, we then obtain

$$\begin{aligned}&\left\langle G^{(\ell _m)}_{\lambda ,m}\left| \left[ (-i \nabla \!+\! \textbf{A}_m)^2 + \lambda ^2 \right] e^{i \textbf{S}_{m}(\textbf{x}_m) \cdot (\textbf{x} - \textbf{x}_m)}\, \xi _{m} \phi _{2,\lambda }\right. \right\rangle \nonumber \\&\quad = \tfrac{\Gamma \left(|\ell _m + \alpha _m|\right)}{ 2^{1 - |\ell _m + \alpha _m|}} \lim _{r \rightarrow 0^{+}} \tfrac{1}{r^{|\ell _m + \alpha _m|}} \int _{0}^{2\pi }\!\! \textrm{d}\theta \; \tfrac{e^{-i\, \ell _m \theta }}{\sqrt{2\pi }} \Big ( r\,\partial _r \phi _{2,\lambda } + |\ell _m + \alpha _m|\, \phi _{2,\lambda } \Big ) \,. \end{aligned}$$

Summing up, the above results entail

$$\begin{aligned}&\sum \limits _{n,\ell _n'} q^{(\ell _n')}_{2,n} \left[B^{(\ell _m \ell _n')}_{m\,n} + \tfrac{\pi ^2\,\lambda ^{2|\ell _n + \alpha _n|}}{\sin (\pi \alpha _n)}\,\delta _{mn}\,\delta _{\ell _m \ell _n^{\prime }} \right] \\&\quad = \lim _{r \rightarrow 0^{+}} \tfrac{\pi \, 2^{|\ell _m + \alpha _m|} \,\Gamma \big (|\ell _m + \alpha _m|\big )}{r^{|\ell _m + \alpha _m|}} \left\langle \Big ( r\,\partial _r \phi _{2,\lambda } + |\ell _m + \alpha _m|\, \phi _{2,\lambda } \Big ) \tfrac{e^{-i\, \ell _m \theta }}{\sqrt{2\pi }} \right\rangle _{m} , \end{aligned}$$

which completes the derivation of the domain (1.23). $\square$

### Singular Perturbations: Kreǐn Theory

Building on the results derived in the previous section and using the general theory described in [66], we henceforth present an equivalent characterization of the Hamiltonian operators $H_{N}^{(B)}$ in terms of singular perturbations of the Friedrichs Hamiltonian $H_{N}^{(\textrm{F})}$. Fixing arbitrarily $z_0 \in \mathbb {C} {\setminus } [0,+\infty )$, we recall (1.29):

$$\begin{aligned} \Lambda (z):= \varvec{\tau } \left( \tfrac{1}{2} \left( \mathcal {G}(z_0) + \mathcal {G}(\bar{z}_0) \right) - \mathcal {G}(z) \right): \mathbb {C}^{2N} \rightarrow \mathbb {C}^{2N}. \end{aligned}$$

Abstract resolvent arguments grant the well posedness of $\Lambda (z)$ and further ensure the following, for all $z,w \in \mathbb {C} {\setminus } [0,+\infty )$ (see [66, Lemma 2.2] and the related discussion):

2.21 $$\begin{aligned} \Lambda (z) - \Lambda (w) = (z-w) \,\breve{\mathcal {G}}(w)\, \mathcal {G}(z), \qquad \big (\Lambda (z)\big )^{*} = \Lambda (z^{*}). \end{aligned}$$

Keeping in mind that $H_{N}^{(\textrm{F})}$ is bounded from below, for any Hermitian matrix $\Theta \in M_{2N,\,\textrm{Herm}}(\mathbb {C})$ there certainly exists a non-empty open set $\mathcal {Z} \subset \mathbb {C} {\setminus } [0,+\infty )$ such that $\Theta + \Lambda (z)$ is invertible for all $z \in \mathcal {Z}$. Taking this into account, from [66, Theorem 2.1] we deduce the following.

#### Proposition 2.10

(Singular perturbations of $R_N^{(\textrm{F})}$). For any $z \in \mathcal {Z}$, the bounded operator

$$\begin{aligned} R_N^{(\Theta )}(z):= R_N^{(\textrm{F})}(z) + \mathcal {G}(z) \big [ \Theta + \Lambda (z) \big ]^{-1} \breve{\mathcal {G}}(z), \end{aligned}$$

is the resolvent of the self-adjoint operator $H_{N}^{(\Theta )}$ coinciding with $H_{N}^{(\textrm{F})}$ on $\ker \varvec{\tau }$ and defined by (1.27), i.e.,

We next prove that the self-adjoint operators $H_{N}^{(\Theta )}$ defined in Theorem 2.10 are nothing but a reparametrization of the Hamiltonians $H_{N}^{(B)}$ discussed previously in Theorem 1.4.

#### Proposition 2.11

For any $\lambda _0 > 0$ large enough, there is a one-to-one correspondence between the families of self-adjoint realizations $\left\{ H_{N}^{(\Theta )} \right\} _{\Theta \in M_{2N,\,\text {Herm}}(\mathbb {C})}$ and $\left\{ H_{N}^{(B)} \right\} _{B \in M_{2N,\,\text {Herm}}(\mathbb {C})}$, which is realized by

2.22 $$\begin{aligned} \Theta _{mn}^{\ell _m \ell _n'}&= B^{(\ell _m \ell _n')}_{m\,n} + \tfrac{\pi \,\lambda _0^{2|\ell _n + \alpha _n|}}{2 \sin (\pi \alpha _n)}\,\delta _{mn}\,\delta _{\ell _m \ell _n'} \nonumber \\ {}&\quad + \left. \left\langle e^{- i {\textbf {S}}_{m}({\textbf {x}}_m) \cdot ({\textbf {x}} - {\textbf {x}}_m)} P_m\, G_{\lambda _0,m}^{(\ell _m)} \right| \right. \nonumber \\ {}&\quad \left| \left. \big [1 + T^{*}_N\big (-\lambda _0^2\big )\big ]^{-1} e^{-i {\textbf {S}}_{n}({\textbf {x}}_n) \cdot ({\textbf {x}} - {\textbf {x}}_n)} \xi _{n} G^{(\ell _n')}_{\lambda _0,n} \right. \right\rangle . \end{aligned}$$

Notice that the relation (2.22) cannot be explicitly resolved to express $\Theta$ in terms of *B*, due to the presence of the unkown inverse operator $[1+T^{*}_N(-\lambda _0^2)]^{-1}$ in the expectation on the r.h.s.. As a consequence, the parametrization of the self-adjoint extensions in terms of the matrix $\Theta$ does not allow to write an explicit relation between the coefficients of the singular and regular terms in the local expansion close the fluxes of the wave functions in the domain (see Remark 1.6 and (1.25)).

Before discussing the proof of the above result, we need to state some technical lemmas. Making reference to Proposition 2.1, we henceforth take $\lambda , \lambda _0 > 0$ large enough so that

2.23 $$\begin{aligned} \big \Vert T_N\big (\!-\lambda ^2\big )\big \Vert< 1,\qquad \big \Vert T_N\big (\!-\lambda _0^2\big )\big \Vert < 1 \end{aligned}$$

This choice of $\lambda , \lambda _0$ ensures the well posedness of the representation (1.30) for the Friedrichs resolvent in both $z = - \lambda ^2$ and $z_0: = - \lambda _0^2$.

#### Lemma 2.12

For any $\lambda > 0$ fulfilling (2.23) and for all $\psi \in L^2(\mathbb {R}^2)$, $\textbf{q} \in \mathbb {C}^{2N}$ there holds:

2.24 $$\begin{aligned}&\breve{\mathcal {G}}(-\lambda ^2) \psi = \textstyle \bigoplus _{n,\ell _n}\! \left.\left\langle \big [1 \!+\! T^{*}_N\big (\!-\lambda ^2\big )\big ]^{-1} e^{-i \textbf{S}_{n}(\textbf{x}_n) \cdot (\textbf{x} - \textbf{x}_n)} \xi _{n} G^{(\ell _n)}_{\lambda ,n} \right| \psi \right\rangle \,; \end{aligned}$$

2.25 $$\begin{aligned}&\mathcal {G}(-\lambda ^2) \textbf{q} = \big [1 \!+\! T^{*}_N\big (\!-\lambda ^2\big )\big ]^{-1} \sum \limits _{n,\ell _n} q_n^{(\ell _n)} e^{-i \textbf{S}_{n}(\textbf{x}_n) \cdot (\textbf{x} - \textbf{x}_n)} \xi _{n} G^{(\ell _n)}_{\lambda ,n} \,. \end{aligned}$$

Moreover, for any fixed $\lambda _0 > 0$ fulfilling (2.23), there holds

2.26 $$\begin{aligned}&\left[\Lambda \big (\!-\lambda ^2\big )\right]_{mn}^{\ell _m \ell _n'}\! = \tfrac{\pi \,\lambda ^{2|\ell _n + \alpha _n|}}{2 \sin (\pi \alpha _n)}\,\delta _{mn}\delta _{\ell _m \ell _n'} - \tfrac{\pi \,\lambda _0^{2|\ell _n + \alpha _n|}}{ 2 \sin (\pi \alpha _n)}\,\delta _{mn}\delta _{\ell _m \ell _n'} \nonumber \\&\quad + \left\langle e^{- i \textbf{S}_{m}(\textbf{x}_m) \cdot (\textbf{x} - \textbf{x}_m)} P_m\, G_{\lambda ,m}^{(\ell _m)}\left| \big [1 \!+\! T^{*}_N\big (\!-\lambda ^2\big )\big ]^{-1} e^{-i \textbf{S}_{n} (\textbf{x}_n) \cdot (\textbf{x} - \textbf{x}_n)} \xi _{n} G^{(\ell _n')}_{\lambda ,n} \right. \right\rangle \nonumber \\&\quad - \left\langle e^{- i \textbf{S}_{m}(\textbf{x}_m) \cdot (\textbf{x} - \textbf{x}_m)} P_m\, G_{\lambda _0,m}^{(\ell _m)}\left| \big [1 \!+\! T^{*}_N\big (\!-\lambda _0^2\big )\big ]^{-1} e^{-i \textbf{S}_{n}(\textbf{x}_n) \cdot (\textbf{x} - \textbf{x}_n)} \xi _{n} G^{(\ell _n')}_{\lambda _0,n} \right. \right\rangle . \end{aligned}$$

#### Proof

We first point out that the expressions in the second and third lines of (2.26) are well defined since

$$\begin{aligned} P_{n}\, G^{(\ell _n)}_{\lambda ,n} \in L^2(\mathbb {R}^2). \end{aligned}$$

Let us further highlight that the second identity in (2.21) grants the self-adjointness of $\Lambda \big (\!-\lambda ^2\big )$, meaning that

$$\begin{aligned} \left[\Lambda \big (\!-\lambda ^2\big )\right]_{mn}^{\ell _m \ell _n'} = \left( {\left[\Lambda \big (\!-\lambda ^2\big )\right]_{n m}^{\ell _n' \ell _m}}\right) ^{\!*}. \end{aligned}$$

As a matter of fact, taking into account that $G^{(\ell _n)}_{\lambda ,n} = \big (\tau ^{(\ell _n)}_{n} R^{(\textrm{F})}_{n}(-\lambda ^2)\big )^{*}$, one gets that the matrix

$$\begin{aligned} \left( \, \left\langle e^{- i \textbf{S}_{m}(\textbf{x}_m) \cdot (\textbf{x} - \textbf{x}_m)} P_m\, G_{\lambda ,m}^{(\ell _m)}\left| \big [1 \!+\! T^{*}_N\big (\!-\lambda ^2\big )\big ]^{-1} e^{-i \textbf{S}_{n}(\textbf{x}_n) \cdot (\textbf{x} - \textbf{x}_n)} \xi _{n} G^{(\ell _n')}_{\lambda ,n} \right. \right\rangle \,\right) ^{\ell _m \ell _n'}_{mn} \end{aligned}$$

is itself Hermitian. Recalling the explicit expression (1.31) for $R^{(\textrm{F})}_{n}(z)$, we get for any $\psi \in L^2(\mathbb {R}^2)$2.27where the last identity follows by comparison with the explicit expression (1.14) for the defect function. Indeed, here we used the Lebesgue dominated convergence theorem and the following asymptotic expansions for the Bessel functions $I_\nu (w),K_\nu (w)$ ($\nu > 0$ and $w> 0$) in the limit $w \rightarrow 0^{+}$ and analogous asymptotics for their derivatives [61, §10.31]:

$$\begin{aligned} I_{\nu }(w)= & {} w^{\nu } \left[ \tfrac{1}{2^{\nu } \Gamma (1 + \nu )} + \tfrac{1}{2^{2 + \nu } \Gamma (2 + nu)}\,w^2 + \mathcal {O}(w^4) \right],\\ K_{\nu }(w)= & {} \tfrac{1}{w^{\nu }} \left[ \tfrac{\Gamma (\nu )}{2^{1-\nu }} - \tfrac{\Gamma (-1 + \nu )}{ 2^{3-\nu }}\, w^2 + \mathcal {O}(w^4) \right] + w^{\nu } \left[ \tfrac{\Gamma (-\nu )}{2^{1+ \nu }} - \tfrac{\Gamma (-1-\nu )}{ 2^{3+\nu }}\, w^2 + \mathcal {O}(w^4) \right]. \end{aligned}$$

Using now the representation (1.30) for the Friedrichs resolvent, we obtain

$$\begin{aligned} \breve{\mathcal {G}}(-\lambda ^2) \psi&=\textstyle \bigoplus _{n,\ell _n} \tau _{n}^{(\ell _n)} R^{(\textrm{F})}_{n}(-\lambda ^2)\, \xi _{n}\, e^{i \textbf{S}_{n}(\textbf{x}_n) \cdot (\textbf{x} - \textbf{x}_n)}\, \big [1 + T_N(-\lambda ^2)\big ]^{-1} \psi \\&= \textstyle \bigoplus _{n,\ell _n}\! \left\langle e^{-i \textbf{S}_{n} (\textbf{x}_n) \cdot (\textbf{x} - \textbf{x}_n)} \xi _{n} G^{(\ell _n)}_{\lambda ,n} \left|\big [1 \!+\! T_N(-\lambda ^2)\big ]^{-1} \psi \right. \right\rangle , \end{aligned}$$

which proves (2.24) for all $\psi \in L^2(\mathbb {R}^2)$. Taking this into account, for any $\textbf{q} \in \mathbb {C}^{2N}$, we get

$$\begin{aligned}&\left.\left\langle \mathcal {G}(-\lambda ^2) \textbf{q}\right|\psi \right\rangle = \left\langle \textbf{q}\left|\breve{\mathcal {G}}(-\lambda ^2) \psi \right. \right\rangle \\&\quad = \sum \limits _{n,\ell _n} \big (q_n^{(\ell _n)}\big )^* \left\langle e^{-i \textbf{S}_{n}(\textbf{x}_n) \cdot (\textbf{x} - \textbf{x}_n)} \xi _{n} G^{(\ell _n)}_{\lambda ,n} \left|\big [\mathbb {1}\!+\! T_N(-\lambda ^2)\big ]^{-1} \psi \right. \right\rangle \\&\quad = \left.\left\langle \big [\mathbb {1}\!+\! T^{*}_N(-\lambda ^2)\big ]^{-1} {\sum \limits _{n,\ell _n}} q_n^{(\ell _n)} e^{-i \textbf{S}_{n}(\textbf{x}_n) \cdot (\textbf{x} - \textbf{x}_n)} \xi _{n} G^{(\ell _n)}_{\lambda ,n} \right|\psi \right\rangle , \end{aligned}$$

which provides evidence for (2.25). To say more, (2.25) can also be rephrased as

2.28 $$\begin{aligned}&\mathcal {G}(-\lambda ^2) \textbf{q} = {\sum \limits _{n,\ell _n}} q_n^{(\ell _n)} e^{-i \textbf{S}_{n}(\textbf{x}_n) \cdot (\textbf{x} - \textbf{x}_n)} \xi _{n} G^{(\ell _n)}_{\lambda ,n} \nonumber \\&- T^{*}_N(-\lambda ^2) \big [1 \!+\! T^{*}_N(-\lambda ^2)\big ]^{-1} {\sum \limits _{n,\ell _n}} q_n^{(\ell _n)} e^{-i \textbf{S}_{n}(\textbf{x}_n) \cdot (\textbf{x} - \textbf{x}_n)} \xi _{n} G^{(\ell _n)}_{\lambda ,n}\,, \end{aligned}$$

and the definition (1.29) yields, for any $\textbf{q} \in \mathbb {C}^{2N}$,

$$\begin{aligned} \sum \limits _{n,\ell _n'} \! \left[\Lambda \big (\!-\lambda ^2\big )\right]_{mn}^{\ell _m \ell _n'} q_{n}^{(\ell _n')} = \tau _{m}^{(\ell _m)}\! \left[ \mathcal {G}\big (\!-\lambda _0^2\big ) \textbf{q} - \mathcal {G}\big (\!-\lambda ^2\big ) \textbf{q} \right] . \end{aligned}$$

On one side, by direct computations we deduce

$$\begin{aligned}&\tau _{m}^{(\ell _m)}\! \left[ \textstyle \sum \limits _{n,\ell _n'} \left\rbrace q_n^{(\ell _n')} e^{-i \textbf{S}_{n}(\textbf{x}_n) \cdot (\textbf{x} - \textbf{x}_n)} \xi _{n} G^{(\ell _n')}_{\lambda _0,n} - q_n^{(\ell _n')} e^{-i \textbf{S}_{n}(\textbf{x}_n) \cdot (\textbf{x} - \textbf{x}_n)} \xi _{n} G^{(\ell _n')}_{\lambda ,n} \right\lbrace \right] \\&\quad = {\textstyle \sum \limits _{\ell _m^{\prime }}} q_m^{(\ell _m^{\prime })}\! \lim _{r \rightarrow 0^{+}} \tfrac{i^{|\ell _m^{\prime } - \ell _m|}\,2^{|\ell _m + \alpha _m|-1} \,\Gamma \big (|\ell _m \!+\! \alpha _m|\big )}{r^{|\ell _m + \alpha _m|}}\; \times \\&\qquad \times \big ( |\ell _m + \alpha _m| + r\,\partial _{r}\big )\! \left[ \left(\lambda _0^{|\ell _m^{\prime } + \alpha _m|} K_{|\ell _m^{\prime } + \alpha _m|}(\lambda _0\,r) - \lambda ^{|\ell _m^{\prime } + \alpha _m|} K_{|\ell _m^{\prime } + \alpha _m|}(\lambda \,r)\right)\right.\\&\qquad \left. J_{|\ell _m^{\prime } - \ell _m|}\big (\,|\textbf{S}_{m}(\textbf{x}_m)|\,r\big ) \right] \\&\quad = \tfrac{\pi }{2\,\sin (\pi \alpha _m)}\,\left(\lambda ^{2|\ell _m + \alpha _m|} - \lambda _0^{2|\ell _m + \alpha _m|} \right) q_m^{(\ell _m)}\,. \end{aligned}$$

On the other side, by (1.32) we get

2.29 $$\begin{aligned} \tau _{m}^{(\ell _m)} T^{*}_N(-\lambda ^2) \phi _{\lambda }= & {} \left\langle G_{\lambda ,m}^{(\ell _m)}\left|P_{m}^{*}\, e^{i \textbf{S}_{m}(\textbf{x}_m) \cdot (\textbf{x} - \textbf{x}_m)} \phi _{\lambda }\right. \right\rangle \nonumber \\= & {} \left.\left\langle e^{- i \textbf{S}_{m}(\textbf{x}_m) \cdot (\textbf{x} - \textbf{x}_m)} P_m\, G_{\lambda ,m}^{(\ell _m)}\right| \phi _{\lambda } \right\rangle . \end{aligned}$$

Summing up, the above arguments and few additional manipulations suffice to prove (2.26). $\square$

Considering the identity (2.28) derived in the previous proof, we introduce the bounded operator

2.30 $$\begin{aligned}{} & {} \mathcal {F}_{\lambda }: \mathbb {C}^{2N} \rightarrow L^2(\mathbb {R}^2),\nonumber \\{} & {} \mathcal {F}_{\lambda } \textbf{q}:= T^{*}_N(-\lambda ^2) \big [1 \!+\! T^{*}_N(-\lambda ^2)\big ]^{-1} \textstyle \sum \limits _{n,\ell _n} q_n^{(\ell _n)} e^{-i \textbf{S}_{n}(\textbf{x}_n) \cdot (\textbf{x} - \textbf{x}_n)} \xi _{n} G^{(\ell _n)}_{\lambda ,n}.\nonumber \\ \end{aligned}$$

In particular, (2.28) reduces to

$$\begin{aligned} \mathcal {G}(-\lambda ^2) \textbf{q} = \sum \limits _{n,\ell _n} q_n^{(\ell _n)} e^{-i \textbf{S}_{n}(\textbf{x}_n) \cdot (\textbf{x} - \textbf{x}_n)} \xi _{n} G^{(\ell _n)}_{\lambda ,n} - \mathcal {F}_{\lambda } \textbf{q}. \end{aligned}$$

#### Lemma 2.13

For any $\lambda > 0$ fulfilling (2.23) and for all $\textbf{q} \in \mathbb {C}^{2N}$, $\mathcal {F}_{\lambda } \textbf{q} \in \mathscr {D}(H_{N}^{(\textrm{F})} )$ and

2.31 $$\begin{aligned} \big ( H_{N}^{(\textrm{F})} + \lambda ^2\big ) \mathcal {F}_{\lambda } \textbf{q} = {\sum \limits _{n,\ell _n}} q_n^{(\ell _n)}\, e^{-i \textbf{S}_{n}(\textbf{x}_n) \cdot (\textbf{x} - \textbf{x}_n)} P_{n} G_{n}^{(\ell _n)}; \end{aligned}$$

2.32 $$\begin{aligned}{} & {} \left[\varvec{\tau } \mathcal {F}_{\lambda } \right]_{m n}^{\ell _m \ell _n'}\nonumber \\{} & {} \quad = \left\langle e^{- i \textbf{S}_{m}(\textbf{x}_m) \cdot (\textbf{x} - \textbf{x}_m)} P_m\, G_{\lambda ,m}^{(\ell _m)}\left| \big [1 \!+\! T^{*}_N(-\lambda ^2)\big ]^{-1} e^{-i \textbf{S}_{n}(\textbf{x}_n) \cdot (\textbf{x} - \textbf{x}_n)} \xi _{n} G^{(\ell _n')}_{\lambda ,n}\right. \right\rangle .\nonumber \\ \end{aligned}$$

#### Proof

In view of (1.30) and (2.27), we deduce

$$\begin{aligned} \mathcal {F}_{\lambda } \textbf{q}&= T^{*}_N(-\lambda ^2) \big [1 \!+\! T^{*}_N(-\lambda ^2)\big ]^{-1}\\&\quad {\sum \limits _{n,\ell _n}} e^{-i \textbf{S}_{n}(\textbf{x}_n) \cdot (\textbf{x} - \textbf{x}_n)} \xi _{n} R^{(\textrm{F})}_{n}(-\lambda ^2) \xi _{n} e^{i \textbf{S}_{n}(\textbf{x}_n) \cdot (\textbf{x} - \textbf{x}_n)} \big (\tau ^{(\ell _n)}_{n}\big )^{*} q_{n}^{(\ell _n)} \\&= T^{*}_N(-\lambda ^2)\, R^{(\textrm{F})}_{N}(-\lambda ^2) {\sum \limits _{n,\ell _n}} \big (\tau ^{(\ell _n)}_{n}\big )^{*} q_{n}^{(\ell _n)}. \end{aligned}$$

Notice that a direct computation gives $(H^{(\textrm{F})}_{N} + \lambda ^2)\, T_{N}^{*}(-\lambda ^2) = T_{N}(-\lambda ^2)\,(H^{(\textrm{F})}_{N} + \lambda ^2)$, which in turn implies

$$\begin{aligned} T_{N}^{*}(-\lambda ^2)\,R^{(\textrm{F})}_{N}(-\lambda ^2) = R^{(\textrm{F})}_{N}(-\lambda ^2)\, T_{N}(-\lambda ^2) . \end{aligned}$$

Taking this into account and using (2.29), for any $\phi \in L^2(\mathbb {R}^2)$ we obtain

$$\begin{aligned} \left.\left\langle \mathcal {F}_{\lambda } \textbf{q}\right| \phi \right\rangle&= {\sum \limits _{n,\ell _n}}\! \left.\left\langle R^{(\textrm{F})}_{N}(-\lambda ^2) T_N(-\lambda ^2) \big (\tau ^{(\ell _n)}_{n}\big )^{*} q_{n}^{(\ell _n)}\right| \phi \right\rangle \\&= {\sum \limits _{n,\ell _n}} \big ({q_{n}^{(\ell _n)}}\big )^* \, \tau ^{(\ell _n)}_{n} T^{*}_N(-\lambda ^2) R^{(\textrm{F})}_{N}(-\lambda ^2) \phi \\&= {\sum \limits _{n,\ell _n}} \big ({q_{n}^{(\ell _n)}}\big )^*\! \left.\left\langle e^{- i \textbf{S}_{n}(\textbf{x}_n) \cdot (\textbf{x} - \textbf{x}_n)} P_n\, G_{\lambda ,n}^{(\ell _n)}\right| R^{(\textrm{F})}_{N}(-\lambda ^2) \phi \right\rangle , \end{aligned}$$

which entails

$$\begin{aligned} \mathcal {F}_{\lambda } \textbf{q} = R^{(\textrm{F})}_{N}(-\lambda ^2)\; { \sum \limits _{n,\ell _n}} e^{- i \textbf{S}_{n}(\textbf{x}_n) \cdot (\textbf{x} - \textbf{x}_n)} P_n\, G_{\lambda ,n}^{(\ell _n)} q_{n}^{(\ell _n)}. \end{aligned}$$

This proves (2.31) and the fact that $\mathcal {F}_{\lambda } \textbf{q}$ belongs to the domain of the Friedrichs realization. The identity (2.32) follows straightforwardly from (2.29) and (2.30).

$$\square $$

In view of Lemma 2.13, it is natural to wonder how the Hamiltonian operators $H_{N}^{(B)}$ and $H_{N}^{(\Theta )}$ are related.

#### Proof of Proposition 2.11

We prove the statement showing that $\mathscr {D}\big (H_{N}^{(\Theta (B))}\big ) = \mathscr {D}\big (H_{N}^{(B)}\big )$, where $\Theta (B)$ is the map (2.22), and $H_{N}^{(\Theta (B))} \psi = H_{N}^{(B)} \psi$, for any $\psi \in \mathscr {D}\big (H_{N}^{(\Theta (B))}\big )$. On the one hand, by Theorem 1.4, for any $\psi \in \mathscr {D}\big (H_{N}^{(B)}\big )$ there exist $\phi _{\lambda } \in \mathscr {D}\big (H_{N}^{(\textrm{F})}\big )$ and $\textbf{q} \in \mathbb {C}^{2N}$ such that

$$\begin{aligned}{} & {} \psi = \phi _{\lambda } + {\sum \limits _{n,\ell _n}}\, q^{(\ell _n)}_{n}\, e^{-i \textbf{S}_{n}(\textbf{x}_n) \cdot (\textbf{x} - \textbf{x}_n)}\, \xi _{n}\, G_{\lambda ,n}^{(\ell _n)};\\{} & {} \tau _{m}^{(\ell _m)}\phi _{\lambda } = \sum \limits _{n,\ell _n} \left[B^{(\ell _m \ell _n)}_{m\,n} + \tfrac{\pi \,\lambda ^{2|\ell _n + \alpha _n|}}{ 2\sin (\pi \alpha _n)}\,\delta _{mn}\,\delta _{\ell _n \ell _n^{\prime }} \right] q^{(\ell _n)}_{n}; \\{} & {} \big ( H_{N}^{(B)} + \lambda ^2\big ) \psi = \big ( H_{N}^{(\textrm{F})} + \lambda ^2\big ) \phi _{\lambda } + \textstyle \sum \limits _{n,\ell _n}\! q^{(\ell _n)}_{n}\, e^{-i \textbf{S}_{n}(\textbf{x}_n) \cdot (\textbf{x} - \textbf{x}_n)}\, P_{n}\,G^{(\ell _n)}_{\lambda ,n}. \end{aligned}$$

On the other hand, for any $\psi \in \mathscr {D}\big (H_{N}^{(\Theta )}\big )$, Proposition 2.10 yields that $\psi = \varphi _{\lambda } + \mathcal {G}(-\lambda ^2) \textbf{q}$, for some suitable $\varphi _{\lambda } \in \mathscr {D}\big (H_{N}^{(\textrm{F})}\big )$ and $\textbf{q} \in \mathbb {C}^{2N}$ with

$$\begin{aligned}{} & {} \tau _{m}^{(\ell _m)} \varphi _{\lambda } = \sum \limits _{n,\ell _n} \big [\Theta _{mn}^{\ell _m \ell _n} + \Lambda _{mn}^{\ell _m \ell _n}(-\lambda ^2)\big ] q_{n}^{(\ell _n)} ; \\{} & {} \big (H_{N}^{(\Theta )} + \lambda ^2 \big ) \psi = \big (H_{N}^{(\textrm{F})} + \lambda ^2\big ) \varphi _\lambda . \end{aligned}$$

In view of Lemma 2.13, we may set

2.33 $$\begin{aligned} \phi _{\lambda } = \varphi _{\lambda } - \mathcal {F}_{\lambda } \textbf{q}. \end{aligned}$$

Then, using the boundary trace operator (1.28), together with the identities (2.26) and (2.32), we deduce

$$\begin{aligned} 0&= \tau _{m}^{(\ell _m)}\phi _{\lambda } - \tau _{m}^{(\ell _m)}\varphi _{\lambda } + \tau _{m}^{(\ell _m)} \mathcal {F}_{\lambda } \textbf{q} \\&= \sum \limits _{n,\ell _n'} \left[B^{(\ell _m \ell _n')}_{m\,n} + \tfrac{\pi \,\lambda ^{2|\ell _n + \alpha _n|}}{ 2\sin (\pi \alpha _n)}\,\delta _{mn}\,\delta _{\ell _m \ell _n^{\prime }} - \Theta _{mn}^{\ell _m \ell _n'} - \Lambda _{mn}^{\ell _m \ell _n'}(-\lambda ^2) \right. \\&\quad \left. + \left\langle e^{- i \textbf{S}_{m}(\textbf{x}_m) \cdot (\textbf{x} - \textbf{x}_m)} P_m\, G_{\lambda ,m}^{(\ell _m)}\left| \big [1 \!+\! T^{*}_N(-\lambda ^2)\big ]^{-1} e^{-i \textbf{S}_{n}(\textbf{x}_n) \cdot (\textbf{x} - \textbf{x}_n)} \xi _{n} G^{(\ell _n^{\prime })}_{\lambda ,n} \right. \right\rangle \right] q^{(\ell _n^{\prime })}_{n} \\&=\sum \limits _{n,\ell _n^{\prime }} \left[ B^{(\ell _m \ell _n')}_{m\,n} - \Theta _{mn}^{\ell _m \ell _n'} + \tfrac{\pi \,\lambda _0^{2|\ell _n + \alpha _n|}}{ 2 \sin (\pi \alpha _n)}\,\delta _{mn}\delta _{\ell _m \ell _n^{\prime }} \right. \\&\qquad \left. + \left\langle e^{- i \textbf{S}_{m}(\textbf{x}_m) \cdot (\textbf{x} - \textbf{x}_m)} P_m\, G_{\lambda _0,m}^{(\ell _m)}\left| \big [1 \!+\! T^{*}_N\big (-\lambda _0^2\big )\big ]^{-1} e^{-i \textbf{S}_{n}(\textbf{x}_n) \cdot (\textbf{x} - \textbf{x}_n)} \xi _{n} G^{(\ell _n^{\prime })}_{\lambda _0,n} \right. \right\rangle \right] q^{(\ell _n^{\prime })}_{n} \,. \end{aligned}$$

In view of the arbitrariness of the charge $\textbf{q} \in \mathbb {C}^{2N}$, the above condition can be fulfilled only by fixing the Hermitian matrix $\Theta$ as in (2.22). To say more, with the position (2.33), by means of (2.31) we obtain

$$\begin{aligned} \big ( H_{N}^{(B)} + \lambda ^2\big ) \psi&= \big ( H_{N}^{(\textrm{F})} + \lambda ^2\big ) \varphi _{\lambda } - \big ( H_{N}^{(\textrm{F})} + \lambda ^2\big )\mathcal {F}_{\lambda } q\\&\quad + \sum \limits _{n,\ell _n}\! q^{(\ell _n)}_{n}\, e^{-i \textbf{S}_{n}(\textbf{x}_n) \cdot (\textbf{x} - \textbf{x}_n)}\, P_{n}\,G^{(\ell _n)}_{\lambda ,n} \nonumber \\&= \big ( H_{N}^{(\textrm{F})} + \lambda ^2\big ) \varphi _{\lambda } = \big (H_{N}^{(\Theta (B))} + \lambda ^2 \big ) \psi \,, \end{aligned}$$

which concludes the proof. $\square$

### Completion of the Proofs

We are finally in position to prove the main results about the singular realizations of the Schrödinger operator $H_N$. Let us first complete the proof of Theorem 1.4.

#### Proof of Theorem 1.4—Part II

It just remains to show that the family of operators $H_N^{(B)}$, with $B \in M_{2N,\,\text {Herm}}(\mathbb {C}) \cup \left\{ \infty \right\}$, exhausts all admissible self-adjoint realizations of the Schrödinger operator $H_{N}$. This is, however, a straightforward consequence of Proposition 2.10, Proposition 2.11 and [68, Theorem 3.1 and Corollary 3.2]. (Arguably, the same conclusion could also be derived adapting the arguments presented in [19] or in [16, Theorem 2.5].) $\square$

#### Proof of Proposition 1.8

Proposition 2.10 and Proposition 2.11 ensure that the perturbed resolvent $R^{(B)}_{N}(z)$ is a finite range perturbation of the Friedrichs analogue $R^{(\textrm{F})}_{N}(z)$. Then, $R^{(B)}_{N}(z) - R^{(\textrm{F})}_{N}(z)$ is trace class and the statement follows by Kuroda–Birman theorem [69, Thm. XI.9] (see also [44]).

To say more, from [67, Theorem 3.4] we infer that the map $\textbf{q} \in \mathbb {C}^{2N} \mapsto \mathcal {G}(-\lambda ^2)\,\textbf{q} \in L^2(\mathbb {R}^{2})$ is a bijection from $\ker \big [\Theta (B) + \Lambda (-\lambda ^2) \big ]$ onto $\ker \big (H_{N}^{(B)} + \lambda ^2 \big )$. In other words, for any given negative eigenvalue $-\lambda ^{2} \in \sigma _{\text {disc}}\big (H_{N}^{(B)}\big )$, $\mathcal {G}(-\lambda ^2)\,\textbf{q}$ is an associated eigenvector for any $\textbf{q} \in \ker \big [\Theta (B) + \Lambda (-\lambda ^2) \big ]$. $\square$

#### Proof of Proposition 1.9

The result follows from Proposition 2.4 and the already mentioned Kuroda–Birman theorem, exploiting once more that $R^{(B)}_{N}(z)$ is a finite range perturbation of $R^{(\textrm{F})}_{N}(z)$. $\square$

## Footnotes

1 Here and in the following we denote by *C* a positive finite constant, whose value may change from line to line.
